# Socioeconomic position and the COVID-19 care cascade from testing to mortality in Switzerland: a population-based analysis

**DOI:** 10.1016/S2468-2667(21)00160-2

**Published:** 2021-07-10

**Authors:** Julien Riou, Radoslaw Panczak, Christian L Althaus, Christoph Junker, Damir Perisa, Katrin Schneider, Nicola G Criscuolo, Nicola Low, Matthias Egger

**Affiliations:** a Institute of Social and Preventive Medicine, University of Bern, Bern, Switzerland; b Federal Office of Public Health, Liebefeld, Switzerland; c Department of Environmental Systems Science, ETH Zürich, Zurich, Switzerland; d Population Health Sciences, Bristol Medical School, University of Bristol, Bristol, UK; e Centre for Infectious Disease Epidemiology and Research, University of Cape Town, Cape Town, South Africa

## Abstract

**Background:**

The inverse care law states that disadvantaged populations need more health care than advantaged populations but receive less. Gaps in COVID-19-related health care and infection control are not well understood. We aimed to examine inequalities in health in the care cascade from testing for SARS-CoV-2 to COVID-19-related hospitalisation, intensive care unit (ICU) admission, and death in Switzerland, a wealthy country strongly affected by the pandemic.

**Methods:**

We analysed surveillance data reported to the Swiss Federal Office of Public Health from March 1, 2020, to April 16, 2021, and 2018 population data. We geocoded residential addresses of notifications to identify the Swiss neighbourhood index of socioeconomic position (Swiss-SEP). The index describes 1·27 million small neighbourhoods of approximately 50 households each on the basis of rent per m^2^, education and occupation of household heads, and crowding. We used negative binomial regression models to calculate incidence rate ratios (IRRs) with 95% credible intervals (CrIs) of the association between ten groups of the Swiss-SEP index defined by deciles (1=lowest, 10=highest) and outcomes. Models were adjusted for sex, age, canton, and wave of the epidemic (before or after June 8, 2020). We used three different denominators: the general population, the number of tests, and the number of positive tests.

**Findings:**

Analyses were based on 4 129 636 tests, 609 782 positive tests, 26 143 hospitalisations, 2432 ICU admissions, 9383 deaths, and 8 221 406 residents. Comparing the highest with the lowest Swiss-SEP group and using the general population as the denominator, more tests were done among people living in neighbourhoods of highest SEP compared with lowest SEP (adjusted IRR 1·18 [95% CrI 1·02–1·36]). Among tested people, test positivity was lower (0·75 [0·69–0·81]) in neighbourhoods of highest SEP than of lowest SEP. Among people testing positive, the adjusted IRR was 0·68 (0·62–0·74) for hospitalisation, was 0·54 (0·43–0·70) for ICU admission, and 0·86 (0·76–0·99) for death. The associations between neighbourhood SEP and outcomes were stronger in younger age groups and we found heterogeneity between areas.

**Interpretation:**

The inverse care law and socioeconomic inequalities were evident in Switzerland during the COVID-19 epidemic. People living in neighbourhoods of low SEP were less likely to be tested but more likely to test positive, be admitted to hospital, or die, compared with those in areas of high SEP. It is essential to continue to monitor testing for SARS-CoV-2, access and uptake of COVID-19 vaccination and outcomes of COVID-19. Governments and health-care systems should address this pandemic of inequality by taking measures to reduce health inequalities in response to the SARS-CoV-2 pandemic.

**Funding:**

Swiss Federal Office of Public Health, Swiss National Science Foundation, EU Horizon 2020, Branco Weiss Foundation.

## Introduction

The pandemic of SARS-CoV-2 infections has created unprecedented challenges for society and health-care systems worldwide. Europe has been heavily affected by the pandemic, with over 55 million confirmed cases and over 1·1 million deaths as of mid-June, 2021, .^1^ Compared with neighbouring countries, Switzerland had a high rate of confirmed COVID-19 cases, higher than those of Austria and Italy, and almost double the rate in Germany.^2^ Similarly, there was substantial excess mortality in Switzerland during the first wave and the highest excess mortality among neighbouring countries during the second wave.^3^

Published in 1971, the inverse care law states that “the availability of good medical care tends to vary inversely with the need for it in the population served.”^4^, ^5^ Inequalities in health are a concern in many regions, including in Europe.^6^ In Switzerland, life expectancy varies between neighbourhoods, depending on the neighbourhood's socioeconomic position (SEP).^7^ Health inequalities and inequities might also influence the outcomes of the COVID-19 pandemic.^8^ A study in Massachusetts, USA, SARS-CoV-2 testing resources had been disproportionately allocated to more affluent communities. In the UK Biobank cohort, testing positive for SARS-CoV-2 was related to area-level socioeconomic deprivation, lower educational level, and non-White ethnicity.^9^ The REal-time Assessment of Community Transmission-2 (REACT-2) study, in England, showed a higher prevalence of people with SARS-CoV-2 antibodies in neighbourhoods with high levels of social disadvantage and among minority ethnic communities.^10^ Studies in the USA showed that patients from neighbourhoods or counties with lower median income or higher deprivation were more likely to require intensive care, and more likely to die from COVID-19.^11^, ^12^

**Research in context**

**Evidence before this study**

Mounting evidence exists that the COVID-19 pandemic increased socioeconomic inequalities. We used the COAP Living Evidence on COVID-19 platform to identify relevant literature. This data platform gathers articles and preprints reporting COVID-19-related research from PubMed and preprint servers from Jan 1, 2020, onwards. On April 29, 2021, we searched the platform using the terms (socioeconomic status) OR (socioeconomic position) OR (inequalities) OR (disparities) AND (infection) OR (testing) OR (hospitalisation) OR (hospitalization) OR (death) OR (mortality). We identified published papers and preprints from China, India, Europe, North America, and Latin America. Most researchers analysed geographically aggregated data and focused on the effect of socioeconomic position (SEP) on the number of COVID-19 cases or deaths, at the level of counties or districts, using data from the first wave in spring 2020. These studies showed that rates of reported COVID-19 cases were associated with populations living in areas of lower SEP, who were more mobile than those from more affluent areas. Few studies examined testing patterns for SARS-CoV-2 testing. One study showed that, in Massachusetts, USA, testing resources had been disproportionately allocated to more affluent communities than poorer communities. A study in the city of Santiago, Chile, showed that the association between testing intensity and higher SEP reversed, with more tests in the most affected areas of lower SEP later in the wave. No study examined the nationwide effect of SEP at high spatial resolution and along the entire care cascade.

**Added value of this study**

This study took advantage of national surveillance data covering the whole cascade from testing for SARS-CoV-2 to the need for hospital care and death in Switzerland. Most notifications could be geocoded and linked to the Swiss neighbourhood index of SEP, resulting in large sample sizes. The data showed that people living in areas of higher SEP were more likely to get tested for SARS-CoV-2 but less likely to test positive, be admitted to hospital or the intensive care unit, and less likely to die, compared with those in areas of lower SEP. In the unique setting of a pandemic, this study illustrates the inverse care law (the availability of good medical care tends to vary inversely with the need for it in the population served), which Julian Tudor Hart formulated 50 years ago. The analysis used data up to mid-April, 2021, and thus covered the first and second waves of the pandemic in 2020 and the subsequent increase in the number of cases observed since mid-February, 2021. Analyses used state-of-the-art statistical methods and three different denominators: the general population, the total number of tests, and the number of positive tests. Results were consistent across these denominators.

**Implications of all the available evidence**

Taken together, the evidence shows that the inverse care law and socioeconomic inequalities manifested themselves during the COVID-19 epidemic, both in wealthy Switzerland and lower-income countries. The pandemic has accentuated socioeconomic inequalities in health in many countries. Analyses incorporating COVID-19 surveillance data with publicly available census data can identify the communities and neighbourhoods most affected by the pandemic. The public health response to COVID-19 should address the socioeconomic constraints on following physical distancing rules, isolation, and quarantine. The design of information campaigns and testing and vaccination programmes should take variation in social, spatial, and digital access into account to minimise inequalities in outcomes. Governments and health-care systems should include measures to reduce health inequalities in their preparedness plans for future pandemics.

Inequalities and inequities in health care and infection control should be described and documented at the population level along the COVID-19 cascade—ie, from testing and testing positive to medical care and clinical outcomes. We analysed nationwide, population-based surveillance data from the Swiss Federal Office of Public Health (SFOPH) to examine the association of neighbourhood SEP with testing for SARS-CoV-2, testing positive, hospitalisation, intensive care unit (ICU) admission, and death.

## Methods

### Data sources

In this population-based study of surveillance data, we used mandatory notifications for negative and positive SARS-CoV-2 tests, and for laboratory-confirmed hospitalisations and deaths related to COVID-19, received at the SFOPH until April 14, 2021.^13^ PCR testing capacity was low during the first wave and tests were mainly used for hospitalised patients with severe symptoms compatible with COVID-19. Since June 24, 2020, the Swiss Federal Government has covered the costs of PCR tests, including for people with mild symptoms, those who were notified by the digital contact tracing app, SwissCovid, and people who were asked by health authorities to get tested following close contact with an infected person. Additionally, on Jan 27, 2021, the Federal Government expanded the criteria for reimbursement by covering the costs of tests in people without symptoms, and on March 12, 2021, by covering up to five rapid tests per month. We included records with a date after Feb 29, 2020 (May 22, 2020, for negative tests), from Swiss residents. We excluded notifications with missing or invalid information on age, sex, or place of residence, and duplicate notifications. We geocoded the residential address using geocoded general population data from the Swiss Federal Statistical Office (2018 edition).^14^ We used population data from 2018 and the directory of retirement and nursing homes to identify individuals living in such institutions.^15^

### Index of neighbourhood SEP

The Swiss neighbourhood index of SEP (Swiss-SEP) is based on the national house-to-house census from 2000.^16^ Swiss-SEP includes 1·27 million neighbourhoods of approximately 50 households each, centred on the individual's residential building, with overlapping boundaries. The index uses the median rent per m^2^, the proportion of households headed by a person with primary education or less, the proportion headed by a person in manual or unskilled occupation, and the mean number of people per room (crowding) to characterise neighbourhoods. No data on household income are collected in the Swiss census. The index was constructed using principal component analysis and validated using independent data on households' financial situation^16^ and was standardised to range from 0 (lowest SEP) to 100 (highest SEP).

### Geocoding and linkage to Swiss-SEP

Geocoding of the residential addresses was done using the publicly available data from the Swiss Federal Office of Topography or, in a few cases, using Google Maps Geocoding API. Swiss-SEP index values were aggregated into ten groups using deciles as cutoffs. Where only a postcode was available, we used the Swiss-SEP value corresponding to the centroid of the area. Data were aggregated by canton (26 groups), sex (two groups), age (nine groups: 10-year groups from 0 years to 79 years and ≥80 years), Swiss-SEP (ten groups), and epidemic wave (two groups: before June 8, 2020 [14 weeks], or from June 8, 2020, onwards [35 weeks]) at the SFOPH. June 8, 2020, was the beginning of the first week after the nadir of case counts. The dataset consisted of aggregated data only. We did this research using surveillance data according to the Swiss law on communicable diseases (EpG, SR 818.101). No ethics approval was required.

### Statistical analysis

We examined the association between Swiss-SEP group and counts of SARS-CoV-2 tests, positive tests, hospitalisations, ICU admissions, and deaths in negative binomial regression models to account for unknown overdispersion. We used a Bayesian approach with weakly informative priors to improve the inference in situations with low numbers (eg, deaths in the age group 0–49 years) or multiple interactions. We considered three different denominators: the general population, the total number of tests, and the number of positive tests. Denominators were included as offsets in each model. In a univariate model, we estimated the incidence rate ratio (IRR) with 95% credibility intervals (CrIs) per unit increase in Swiss-SEP group for each outcome and denominator. The model assumes that the association with Swiss-SEP is linear on the logarithmic scale. We tested this assumption by comparing this model with one in which each group was included separately. We estimated the IRR adjusted for age group, sex, canton, and epidemic wave in a second model. The adjustment for canton included a random intercept and slope by canton, allowing for interaction between Swiss-SEP group and canton. In a third model, we assessed two-way interactions between Swiss-SEP, and age group, sex, and epidemic wave. We used the leave-one-out information criterion for model selection.^17^

In sensitivity analyses, we (1) excluded all cases geocoded with the postal code and not the full address and (2) excluded cases with an address corresponding to one of 1586 retirement or nursing homes. All analyses were done using Stan (version 2.21.1)^18^ and R (version 4.0.4), with package rstanarm. We used weakly informative prior distributions for all model parameters.^17^ Calculations were done on UBELIX. The appendix (p 4) provides more information.

### Role of the funding source

The funder of the study had no role in study design, data collection, data analysis, data interpretation, or writing of the report.

## Results

**Figure 1:**
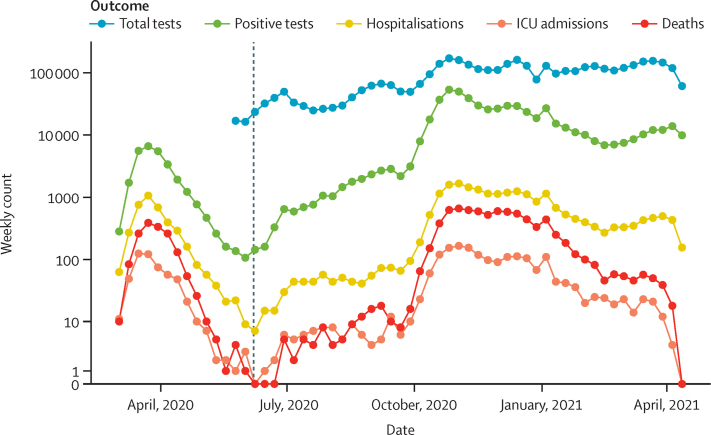
Evolution of notifications to the Federal Office of Public Health during the COVID-19 pandemic in Switzerland from March 1, 2020, to April 14, 2021 The counts of total tests were available only from May 23, 2020. The dashed line shows the date chosen for the separation between the first and second wave (June 8, 2020). ICU=intensive care unit.

As of April 14, 2021, the SFOPH received 6 872 353 notifications related to COVID-19 during two epidemic waves (figure 1). 5 910 732 SARS-CoV-2 test res-ults, 616 239 (10·4%) positive SARS-CoV-2 tests, 26 373 COVID-19 hospital admissions (4·3% of positive tests), 2458 COVID-19 ICU admissions (0·4% of positive cases), and 9550 deaths from COVID-19 (for a case fatality rate of 1·5%) met eligibility criteria (appendix p 2). Valid information on age, sex, and place of residence was available for 4 129 636 (69·9%) tests, 609 782 (99·0%) positive tests, 26 143 (99·1%) hospitalisations, 2432 (98·9%) ICU admissions, and 9383 (98·3%) deaths. In approximately 95% of geocoded notifications, geocoding was based on the exact address (appendix p 3). Few geocodes corresponded to retirement or nursing homes, ranging from 63 (2·1%) of 2432 people admitted to ICU to 1194 (4·6%) of 26 143 people admitted to hospital. For deaths, 3178 notifications (33·9%) were from such retirement or nursing homes (appendix p 3).

Table 1 shows the observed distribution of geocoded notifications and the 2018 general population across age, sex, epidemic wave, and Swiss-SEP group. Approximately 40% of the Swiss population is aged 50 years or older. This age group accounted for 33·0% of all SARS-CoV-2 tests and for 38·9% of positive tests, but for 88·2% of hospitalisations, 92·8% of ICU admissions and 99·6% of deaths (table 1). Women contributed more tests and more positive tests than men did (table 1). Men accounted for most hospitalisations, ICU admissions, and deaths (table 1). The group of lowest SEP accounted for 82 977 (13·6%) of 609 782 positive tests, 4045 (15·5%) of 26 143 hospitalisations, 398 (16·4%) of 2432 ICU admissions, and 1240 (13·2%) of 9383 deaths, whereas the highest SEP accounted for 43 466 (7·1%) positive tests, 1469 (5·6%) hospitalisations, 119 (4·9%) ICU admissions, and 574 (6·1%) deaths (table 1). In the general population, the number of people living in neighbourhoods of lower SEP is higher than in neighbourhoods of higher position. Most of the data were from the second wave, which lasted longer and was more severe than the first; 7808 (83·2%) deaths were from the second wave (table 1).

**Table 1:** Distribution of study data across age, sex, and neighbourhood index of SEP

		Total tests*	**Positive tests**	**Hospitalisations**	**ICU admissions**	**Deaths**	**Population**
Total		4 129 636	609 782	26 143	2432	9383	8 221 406
Age, years							
	0–9	140 728 (3·4%)	10 384 (1·7%)	267 (1·0%)	11 (0·5%)	2 (0·0%)	850 207 (10·3%)
	10–19	476 206 (11·5%)	57 342 (9·4%)	151 (0·6%)	7 (0·3%)	1 (0·0%)	816 042 (9·9%)
	20–29	746 922 (18·1%)	104 977 (17·2%)	450 (1·7%)	17 (0·7%)	2 (0·0%)	1 017 569 (12·4%)
	30–39	770 815 (18·7%)	102 729 (16·8%)	749 (2·9%)	39 (1·6%)	9 (0·1%)	1 181 147 (14·4%)
	40–49	635 320 (15·4%)	96 941 (15·9%)	1489 (5·7%)	100 (4·1%)	32 (0·3%)	1 170 313 (14·2%)
	50–59	584 916 (14·2%)	100 649 (16·5%)	3180 (12·2%)	309 (12·7%)	162 (1·7%)	1 239 500 (15·1%)
	60–69	343 966 (8·3%)	57 595 (9·4%)	4572 (17·5%)	639 (26·3%)	588 (6·3%)	898 741 (10·9%)
	70–79	217 158 (5·3%)	37 102 (6·1%)	6566 (25·1%)	884 (36·3%)	1882 (20·1%)	675 413 (8·2%)
	≥80	213 605 (5·2%)	42 063 (6·9%)	8719 (33·4%)	426 (17·5%)	6705 (71·5%)	372 474 (4·5%)
Sex							
	Male	1 964 095 (47·6%)	291 218 (47·8%)	14 960 (57·2%)	1773 (72·9%)	5045 (53·8%)	4 081 536 (49·6%)
	Female	2 165 541 (52·4%)	318 564 (52·2%)	11 183 (42·8%)	659 (27·1%)	4338 (46·2%)	4 139 870 (50·4%)
COVID-19 wave							
	First wave†	35 375 (0·9%)	28 018 (4·6%)	3929 (15·0%)	533 (21·9%)	1575 (16·8%)	..
	Second wave‡	4 094 261 (99·1%)	581 764 (95·4%)	22 214 (85·0%)	1899 (78·1%)	7808 (83·2%)	..
Neighbourhood index of SEP (group)							
	1 (lowest)	452 438 (11·0%)	82 977 (13·6%)	4045 (15·5%)	398 (16·4%)	1240 (13·2%)	983 036 (12·0%)
	2	436 720 (10·6%)	71 319 (11·7%)	3366 (12·9%)	354 (14·6%)	1140 (12·1%)	885 376 (10·8%)
	3	410 169 (9·9%)	65 886 (10·8%)	2999 (11·5%)	274 (11·3%)	1042 (11·1%)	842 747 (10·3%)
	4	404 986 (9·8%)	61 757 (10·1%)	2796 (10·7%)	254 (10·4%)	977 (10·4%)	827 400 (10·1%)
	5	408 730 (9·9%)	60 782 (10·0%)	2622 (10·0%)	243 (10·0%)	1033 (11·0%)	825 493 (10·0%)
	6	404 213 (9·8%)	59 654 (9·8%)	2533 (9·7%)	227 (9·3%)	1044 (11·1%)	813 598 (9·9%)
	7	408 996 (9·9%)	56 701 (9·3%)	2289 (8·8%)	193 (7·9%)	825 (8·8%)	809 336 (9·8%)
	8	402 915 (9·8%)	55 093 (9·0%)	2137 (8·2%)	187 (7·7%)	864 (9·2%)	791 945 (9·6%)
	9	392 818 (9·5%)	52 147 (8·6%)	1887 (7·2%)	183 (7·5%)	644 (6·9%)	758 723 (9·2%)
	10 (highest)	407 651 (9·9%)	43 466 (7·1%)	1469 (5·6%)	119 (4·9%)	574 (6·1%)	683 752 (8·3%)

Data are n (%). ICU=intensive care unit. SEP=socioeconomic position.

* Data on total tests relate to the period May 23, 2020, to April 14, 2021, rather than the full study period from March 1, 2020, to April 14, 2021.

† The first wave of infections was before June 8, 2020.

‡ The second wave was from June 8, 2020.

**Figure 2:**
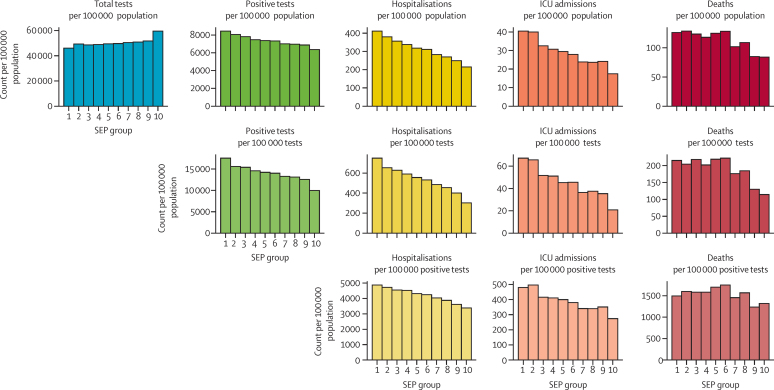
Counts of notified SARS-CoV-2 tests, positive tests, hospitalisations, ICU admissions, and deaths across groups of SEP per 100 000 population, tests, or positive tests Higher SEP groups correspond to neighbourhoods of higher SEP. The study period was March 1, 2020, to April 14, 2021, except for total tests that only covered May 23, 2020, to April 14, 2021. ICU=intensive care unit. SEP=socioeconomic position.

The rates of SARS-CoV-2 tests per population increased with Swiss-SEP group (figure 2), whereas the rates decreased for positive tests, hospitalisations, ICU admissions, and deaths. The slopes for positive tests, hospitalisations, and ICU admissions were steeper when rates were calculated per test rather than by population, and somewhat less steep when expressed per positive test (figure 2).

Modelling Swiss-SEP groups as a continuous variable led to a similar or better model fit than with discrete variables (appendix p 5). Adjusting for age, sex, epidemic wave, and canton improved the fit further (appendix p 5). Visual comparison of model predictions and observed data from the latter model illustrates the good fit, with most observed data points within the 95% CrI of model estimates (appendix p 6). One exception was the high number of tests among people living in neighbourhoods in the highest Swiss-SEP group, which was not captured well. Additionally, several data points were outside the CrI for positive tests per population. The fit improved for positive tests when stratifying the data by epidemic wave (appendix p 14).

**Figure 3:**
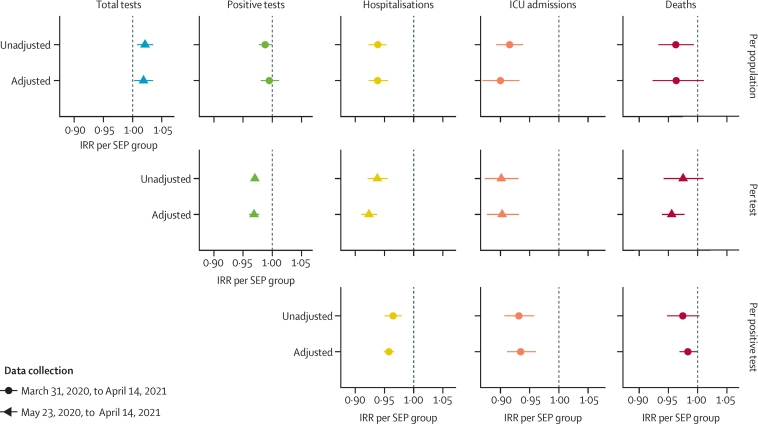
Unadjusted and adjusted IRRs per increase in the group of neighbourhood SEP for the counts of SARS-CoV-2 tests, positive tests, hospitalisations, ICU admissions, and mortality per population, tests or positive tests Median posteriors and 95% credibility intervals are shown in each case. IRR estimates higher than 1 correspond to a positive association with Swiss neighbourhood index of SEP groups; estimates lower than 1 correspond to a negative association. Adjusted estimates are adjusted for age, sex, canton, and epidemic wave. The study period was March 1, 2020, to April 14, 2021, except for total tests, which covered May 23, 2020, to April 14, 2021. ICU=intensive care unit. IRR=incidence rate ratio. SEP=socioeconomic position.

In both unadjusted and adjusted analyses, each increase in Swiss-SEP group was associated with an increase in SARS-CoV-2 testing per population (figure 3). The adjusted IRR was 1·02 (95% CrI 1·00–1·03) per group increase, corresponding to 18% (2–36) more tests in the highest compared with the lowest socioeconomic group (table 2). We did not find any association with positive tests per population (figure 3). The number of positive tests per number of tests decreased as Swiss-SEP group increased (adjusted IRR 0·97 [0·96–0·98]), corresponding to a 25% (19–31) lower test positivity in the highest compared with the lowest socioeconomic group (figure 3). The greater uptake of testing in neighbourhoods of higher socioeconomic position masked the higher number of positive tests among individuals from lower SEP than in higher SEP neighbourhoods. Rates of hospitalisations (adjusted IRR 0·94 [0·92–0·96]) and ICU admissions (0·90 [0·87–0·93] per population decreased with higher SEP, corresponding to 44% (33–51) lower hospitalisation rates and 61% (47–72) lower ICU admission rates in the highest compared with the lowest socioeconomic group (figure 3). Estimates were similar in the unadjusted and adjusted analyses, and similar with different denominators (table 2; figure 3).

**Table 2:** Association of Swiss-SEP group with five outcomes related to SARS-CoV-2 surveillance and care

	**Unadjusted IRR per Swiss-SEP group increase (95% CrI)**	**Adjusted IRR per Swiss-SEP group increase (95% CrI)**	**Adjusted IRR comparing highest and lowest Swiss-SEP group (95% CrI)**
Total tests*			
Per population	1·02 (1·01–1·04)	1·02 (1·00–1·03)	1·18 (1·02–1·36)
**Positive tests**			
Per population	0·99 (0·98–1·00)	0·99 (0·98–1·01)	0·95 (0·84–1·11)
Per total test*	0·97 (0·97–0·98)	0·97 (0·96–0·98)	0·75 (0·69–0·81)
**Hospitalisations**			
Per population	0·94 (0·92–0·95)	0·94 (0·92–0·96)	0·56 (0·49–0·67)
Per total test*	0·94 (0·92–0·95)	0·92 (0·91–0·94)	0·49 (0·43–0·56)
Per positive test	0·96 (0·95–0·98)	0·96 (0·95–0·97)	0·68 (0·62–0·74)
**ICU admissions**			
Per population	0·92 (0·89–0·94)	0·90 (0·87–0·93)	0·39 (0·28–0·53)
Per total test*	0·90 (0·87–0·93)	0·90 (0·88–0·93)	0·40 (0·31–0·53)
Per positive test	0·93 (0·91–0·96)	0·93 (0·91–0·96)	0·54 (0·43–0·70)
**Deaths**			
Per population	0·96 (0·93–0·99)	0·96 (0·92–1·01)	0·71 (0·49–1·10)
Per total test*	0·98 (0·94–1·01)	0·96 (0·94–0·98)	0·66 (0·57–0·82)
Per positive test	0·97 (0·95–1·00)	0·98 (0·97–1·00)	0·86 (0·76–0·99)

Three denominators were considered: population, total tests, and positive tests. CrI=credibility interval. IRR=incidence rate ratio. Swiss-SEP=Swiss neighbourhood index of socioeconomic position.

* Data on total tests relate to the period May 23, 2020, to April 14, 2021, rather than the full study period from March 1, 2020, to April 14, 2021.

**Figure 4:**
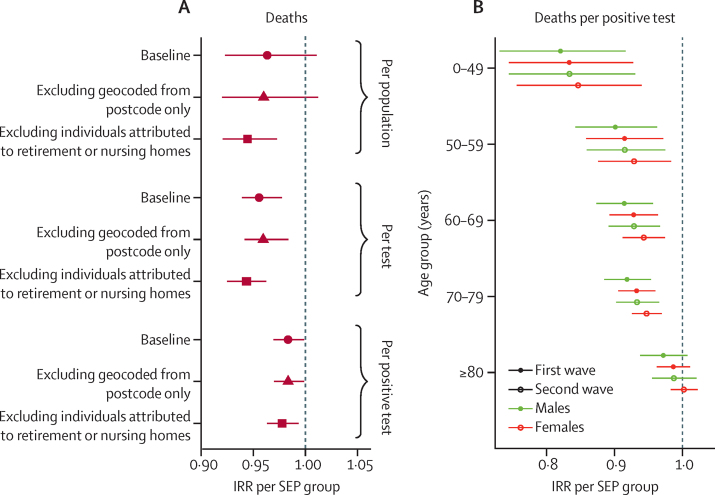
Adjusted IRRs from sensitivity analyses (A) Adjusted IRRs per increase in group of neighbourhood SEP for COVID-19 deaths per population, per test, or per positive test in the baseline analysis or in sensitivity analyses: (1) excluding all cases geocoded from the postcode only and (2) excluding cases with a residential address corresponding to retirement or nursing homes. (B) Adjusted IRRs for COVID-19 deaths per positive test by age group, sex, and epidemic wave. IRR=incidence rate ratio. SEP=socioeconomic position. The first wave of infections was before June 8, 2020, and the second wave from June 8, 2020.

COVID-19-related mortality declined with increasing SEP of neighbourhoods (table 2). The association became stronger when excluding residents of retirement or nursing homes (figure 4A). After excluding such residents, the adjusted IRRs per increase in Swiss-SEP group were 0·94 (95% CrI 0·92–0·97) for COVID-19 deaths per population, 0·94 (0·93–0·96) for deaths among those tested and 0·98 (0·96–0·99) for deaths among those testing positive (figure 4A, appendix p 17). These estimates translated into a lower mortality of 40% (24–52) per population, 40% (29–49) per test, and 18% (7–28) per positive test, comparing the highest with the lowest group (figure 4A).

Rates of testing, positive tests, and clinical outcomes were also associated with age and sex (appendix p 8). The testing rate per 100 000 population was lowest and positive tests were the least frequent in children aged 0–9 years (appendix p 9). The risk of hospitalisation increased with age and ICU admissions and mortality increased from age 50 years onwards. Testing and positive tests were about as frequent among men and women but the rates of hospitalisations, ICU admissions, and mortality were all lower in women than in men. For all outcomes, we found heterogeneity across cantons (appendix p 9).

We examined two-way interactions between Swiss-SEP group and age, sex, epidemic wave, and canton (appendix pp 10–12). The associations between SEP and outcomes became weaker with increasing age (appendix pp 10–12). The interaction with age is illustrated in figure 4B for mortality in those testing positive. The association with neighbourhood SEP became weaker moving from age group 0–49 years to older age groups and disappeared in the age group 80 years and older (appendix pp 10–12). We found little evidence of interactions with sex or epidemic wave. There was also heterogeneity across cantons, particularly for testing and positive tests. The canton of Geneva was an outlier, with a stronger positive association of Swiss-SEP group with testing and a stronger negative association with test positivity compared with the national average. Associations with testing and test positivity were also somewhat stronger for the cantons of Bern, Obwalden, and Uri, and weaker or absent for other cantons (appendix pp 10–12).

## Discussion

In this whole-population study of the COVID-19 epidemic in Switzerland in 2020–21, we found that people living in areas with higher SEPs were more likely to get tested for SARS-CoV-2 but less likely to test positive and be admitted to hospital or the ICU, and less likely to die, compared with those in areas of lower SEP. The strength of the association increased moving along the care cascade from test positivity to hospitalisation and ICU admission. Associations with neighbourhood SEP were similar during the two waves but more pronounced in some areas than others. Testing was less intense and positive tests less frequent in children. The risk of hospitalisation increased continuously with age, and ICU admissions and mortality increased from age 50 years onwards, in line with a previous study.^19^ Testing and positive tests were about as frequent among men and women, but hospitalisations, ICU admissions, and mortality were all higher in men than in women, again confirming previous findings.^20^

We used national data for one country and its health system and covered the cascade from testing for SARS-CoV-2 to testing positive, hospital admission, death, in both waves of the COVID-19 epidemic for all ages. Our study of the Swiss resident population thus avoided the selection bias affecting many COVID-19 studies—eg, studies of patients admitted to hospital.^21^ Another strength is the use of the Swiss-SEP index, which has criterion validity, with mean household income continuously increasing from the lowest to highest SEP group, based on data from more than 1 million small neighbourhoods centred on individuals' residences.^16^

We examined the association with SEP in three different populations: the general population, the population tested for SARS-CoV-2, and the group with positive tests. Associations were consistent across the three populations, except for the rate of positive tests in the population. The greater uptake of testing in higher SEP neighbourhoods masked the higher number of positive tests in those of lower SEP. The data also have weaknesses that limit interpretation. Each outcome is likely to be affected by some level of under-reporting, possibly creating bias if under-reporting is associated with SEP. However, individuals in lower SEP neighbourhoods might be more affected by under-reporting, meaning that our results would underestimate the association between SEP and test positivity, hospital admissions and deaths. The data on tests are limited by the absence of complete data on reasons for testing: the lower test positivity among young children could thus reflect that children were more likely to be tested for infection control rather than because of symptoms, compared with those in older age groups. Alternatively, the lower test positivity could indicate a lower susceptibility to SARS-CoV-2 infection in this age group. Not all notifications could be geocoded because of incomplete addresses. The Swiss-SEP index of retirement and nursing homes might not reflect the neighbourhood where residents spent most of their lives, thus misclassifying their SEP. This limitation might explain why the strength of the association increased when excluding deaths in residents of these institutions. Finally, the Swiss-SEP index is based on the 2000 census, although it continues to be strongly associated with income and mortality.^22^

Data on indicators of SEP are often not collected in clinical studies or routine surveillance systems. Khalatbari-Soltani and colleagues^8^ observed that, up to April, 2020, no study about COVID-19 had reported data on socioeconomic indicators such as educational level, income, or housing conditions.^8^ Since then, several studies have found associations between area-level deprivation and SARS-CoV-2 infection, increased severity of COVID-19 disease, and mortality.^10^, ^11^, ^12^, ^23^, ^24^ In common with these studies, our study used a small area-based measure of SEP. Area-based measures are more readily available than individual measures and have the advantage of capturing effects at the level of both the individual and the place. A seroprevalence study in the canton of Geneva, Switzerland, in 2020, did not find strong associations with individual-level indicators of social position, other than becoming unemployed.^25^ However, the study population was not representative of the population of Geneva, including many more individuals with tertiary education, fewer with mandatory school only, and fewer non-Swiss nationals compared with the general population.^26^ A Swedish study used individual-level data from population-based registers and found that people with lower income and level of education and immigrants from low-income or middle-income countries were at higher risk of death from COVID-19.^27^ Data on ethnicity are not recorded in the Swiss surveillance system, which is a weakness of our study. Of note, the proportion of non-White individuals in the Swiss population is less than 5%. A study of COVID-19 outcomes up to the end of 2020 in 17 million adults in England showed an increased risk of testing positive for SARS-CoV-2 and adverse outcomes in minority ethnic populations.^28^ A strength of both our study and the English study^28^ is that they covered the entire cascade from testing to mortality at the national level. Previous studies were often based on population surveys, with unequal participation across socioeconomic and ethnic groups,^9^, ^10^, ^25^, ^26^ excluded children,^9^, ^10^ or were restricted to selected hospitals, areas, or cities.^11^, ^23^, ^24^

50 years ago, Tudor Hart proposed the inverse care law, which stipulates that “the availability of good medical care tends to vary inversely with the need for it in the population served.”^4^ In 2000, Victora and colleagues^29^ proposed an analogous inverse equity hypothesis, which states that new health interventions are initially adopted by the wealthier segments of a population who have the least need. Our study bears out both hypotheses in the unique setting of a pandemic. Early diagnosis of SARS-CoV-2 infections and adequate initial management might improve the prognosis of individuals with COVID-19, whereas prognosis is worse in patients diagnosed late, with low oxygen saturation and signs of pneumonia.^30^ Rapid diagnosis and isolation are the key to preventing transmission; communities with higher testing levels will benefit from lower rates of transmission. The SARS-CoV-2 tests were a new technology and testing capacity was limited in Switzerland, particularly during the first wave of the pandemic. In both waves, testing was less intense in neighbourhoods of lower SEP. People living in these areas might have had less access to test centres because of reduced access to private transport or an inability to take time off work. Greater availability of testing and conditions that eased uptake in these areas could have improved outcomes and reduced transmission.

The higher rate of positive tests in neighbourhoods of lower SEP might reflect higher risks of SARS-CoV-2 infection at work and at home. People in manual occupations are unable to work from home and are likely to have more unprotected contacts with others, on building sites, or in factories compared with those who could work from home. At home, living conditions might also be more crowded in lower SEP areas than in higher SEP areas. A study from the USA used mobile phone data to show that the adoption of physical distancing was lower in counties with higher proportions of people below the poverty level.^31^ Detailed maps of the SEP of Swiss neighbourhoods have been published.^16^ Health policy measures should consider the susceptibility of different communities and prevent inequities in health and infection control. The Swiss National COVID-19 Science Taskforce recommended that, in this unpredictable crisis, the state should assume the role of insurer and cushion the negative effects with appropriate economic and social policies.^32^ Without such support, those affected will understandably not favour control measures that threaten their livelihoods.

Switzerland is one of the wealthiest countries globally, with wealth more unequally distributed than in other European countries—the Gini coefficient is estimated at 0·86 using 2015 tax data.^33^ Switzerland has a well developed health-care system and universally mandated health insurance, which in principle guarantees access to care for all. The Swiss health-care system has been described as providing a good balance between individual responsibility and community solidarity,^34^ but evidence exists that high out-of-pocket payments, including co-payments and deductibles, might prompt some individuals not to seek care. A survey in the canton of Geneva^35^ showed that up to 31% of respondents reported having foregone health care for economic reasons depending on income. In our study, Geneva was the canton with the strongest association between neighbourhood SEP and testing. Geneva also had the highest Gini index for wealth (0·92).^33^

In conclusion, this nationwide study found that people living in neighbourhoods of higher SEP are more likely to be tested in Switzerland but less likely to test positive, be hospitalised, or die, compared with those living in areas of lower SEP. The higher incidence of SARS-CoV-2 infections, combined with a higher prevalence of comorbidities in neighbourhoods of lower SEP compared with higher SEP is likely to have contributed to worse outcomes, including the higher risk of hospitalisation and death. By June 2021, vaccination coverage had increased considerably, with over 40% of the population having received at least one dose of SARS-CoV 2 vaccine, and the Government is gradually easing preventive measures. It is essential to continue to monitor testing for SARS-CoV-2, access and uptake of COVID-19 vaccination, and outcomes of COVID-19. Governments and health-care systems should address this pandemic of inequality by taking measures to reduce health inequalities in their response to the SARS-CoV-2 pandemic.^36^

## Data sharing

The data are accessible to researchers upon reasonable request for data sharing to the corresponding author. Requests for data need to be approved by the SFOPH. The code is available from https://github.com/jriou/covid-sep-ch.

## Declaration of interests

We declare no competing interests.
