# Supplementary material for: Socioeconomic position and the COVID-19 care cascade from testing to mortality in Switzerland: a population-based analysis
Source: Lancet Public Health. 2021 Jul 10;6(9):e683–91. doi: 10.1016/S2468-2667(21)00160-2 (PMC8270761; doi:10.1016/S2468-2667(21)00160-2)
Supplement: Supplementary appendix [file mmc1.pdf]

THE LANCET
Public Health

Supplementary appendix

This appendix formed part of the original submission and has been peer reviewed. We post it as supplied by the authors.

Supplement to: Riou J, Panczak R, Althaus C L, et al. Socioeconomic position and the COVID-19 care cascade from testing to mortality in Switzerland: a population-based analysis. *Lancet Public Health* 2021; published online July 9. http://dx.doi.org/10.1016/S2468-2667(21)00160-2.

Supplementary appendix to:
From SARS-CoV-2 Testing To COVID-19 Mortality: Analysis of Swiss national surveillance data

Julien Riou[a,b,†], Radoslaw Panczak[a,†], Christian Althaus[a], Christoph Junker[b], Damir Perisa[b], Katrin Schneider[b], Nicola G. Criscuolo[c], Nicola Low[a], and Matthias Egger[a,d,e,*]

[a]Institute of Social and Preventive Medicine, University of Bern, Bern, Switzerland
[b]Federal Office of Public Health, Liebefeld, Switzerland
[c]Department of Environmental Systems Science, ETH Zürich, Zurich, Switzerland
[d]Population Health Sciences, Bristol Medical School, University of Bristol, UK
[e]Centre for Infectious Disease Epidemiology and Research, University of Cape Town, Cape Town, South Africa
[†]contributed equally
[*]Corresponding author (matthias.egger@ispm.unibe.ch)

June 17, 2021

Contents

1 Geocoding procedure **2**
1.1 Included data . . . 2
1.2 Drivers of inclusion . . . 2

2 Extended methods **4**

3 Extended results **4**
3.1 Model selection . . . 4
3.2 Model fit . . . 5
3.3 Socio-economic position . . . 6
3.4 Other covariates . . . 8
3.5 Interaction analysis . . . 10
3.6 Time delays between key events . . . 12

4 Sensitivity analyses **13**
4.1 Stratification by period . . . 13
4.2 Excluding geocodes based only on ZIP code . . . 16
4.3 Excluding geocodes obtained with Google maps . . . 16
4.4 Excluding geocodes attributed to retirement or nursing homes . . . 16

5 Tables of estimates **19**
5.1 Other covariates . . . 19
5.2 Interactions . . . 22

1 Geocoding procedure

1.1 Included data

Geocoding of addresses was done under a contract between the FOPH and the Department of Environmental Sciences at ETH Zürich. Only address data were provided, no other information left the FOPH. Where no address but a ZIP code was available, we used the geocode corresponding to the centroïd of the ZIP code area. We linked each notification to the corresponding value of the Swiss neighbourhood index of SEP, using latitude and longitude of the home address. SEP values were grouped according to deciles. Data were aggregated by canton (26 groups), sex (2 groups), age (9 groups, from 0-9 to 80 and older), SEP decile (10 groups) and epidemic wave (2 groups, before or after 8 June 2020) at the FOPH. The analysis dataset consisted of aggregated data only, and no individual data left the FOPH.

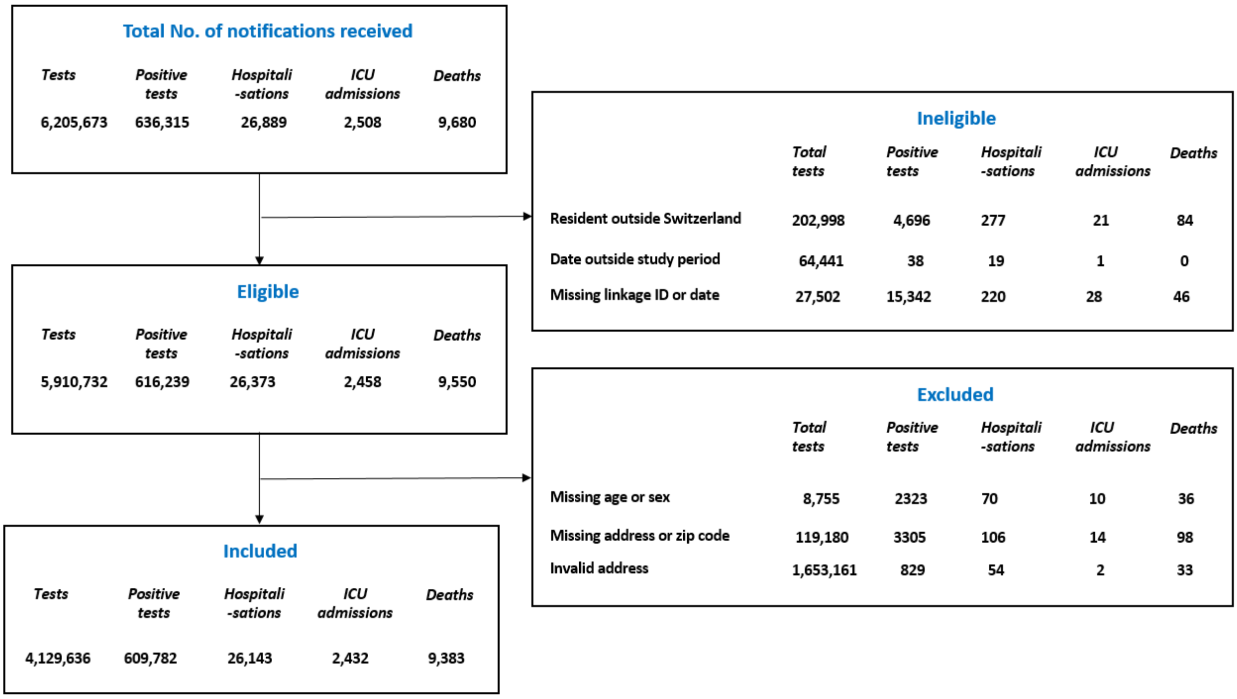

Figure S1: STROBE flowchart.

1.2 Drivers of inclusion

Valid information on age, sex and place of residence was available for 4,129,636 (70%) tests, 609,782 (99%) positive tests, 26,143 (99%) hospitalisations, 2,432 (99%) ICU admissions and 9,383 (98%) deaths. These outcomes were included in the analysis. We investigated the risk factors of inclusion with simple logistic models where age, sex and canton where included as covariates. We found that the probability of inclusion was mostly driven by the canton, suggesting regional differences in data collection and data quality (Figure S2).

Table S1: Details about the geocoding procedure.

	Total tests	Positive tests	Hospitalisations	ICU admissions	Deaths
**All included**	4,129,636	609,782	26,143	2,432	9,383
**Geocoding determined from:**	-	-	-	-	-
full residential addresses	3,981,895 (96.4%)	596,573 (97.8%)	25,511 (97.6%)	2,299 (94.5%)	8,849 (94.3%)
ZIP code only	147,741 (3.6%)	13,209 (2.2%)	632 (2.4%)	133 (5.5%)	534 (5.7%)
**Software used for geocoding:**	-	-	-	-	-
Swisstopo	3,786,463 (91.7%)	539,797 (88.5%)	22,343 (85.5%)	2,072 (85.2%)	7,168 (76.4%)
Google maps	343,173 (8.3%)	69,985 (11.5%)	3,800 (14.5%)	360 (14.8%)	2,215 (23.6%)
**Geocode attributed to retirement and nursing homes:**	-	-	-	-	-
no	4,042,476 (97.9%)	591,147 (96.9%)	24,949 (95.4%)	2,369 (97.4%)	6,205 (66.1%)
yes	87,160 (2.1%)	18,635 (3.1%)	1,194 (4.6%)	63 (2.6%)	3,178 (33.9%)

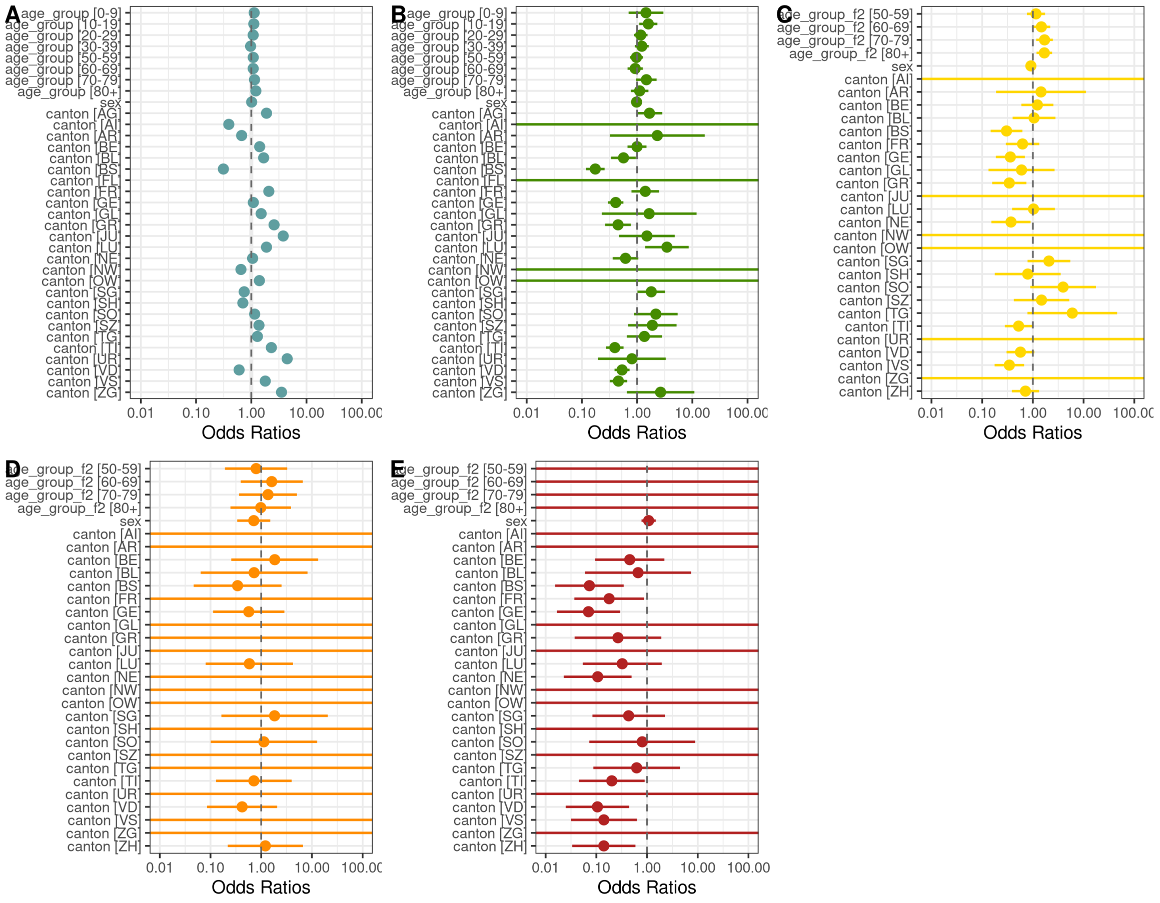

Figure S2: Individual drivers of the probability of inclusion in the analysis for (A) total tests, (B) positive tests, (C) hospitalisations, (D) ICU admissions and (E) deaths. The associations are expressed as adjusted odds-ratios obtained form logistic regression.

2 Extended methods

We analysed the association between SEP decile and counts of 1) SARS-CoV-2 tests, 2) positive cases, 3) COVID-19-related hospitalisations, 4) ICU admissions and 5) deaths with separate negative binomial regression models. The procedure was similar for each of the five outcomes, except in the choice of denominator. Let $y_{i,j}$ be the count of the outcome of interest in a specific combination of SEP decile, age group, sex and period designed as i, and in canton j. There are $10 \times 9 \times 2 \times 2 = 360$ such combinations ($i \in \{1, \dots, 360\}$) in each of the 26 cantons ($j \in \{1, \dots, 26\}$), so $y_{i,j}$ has a total length of 9360. In a first model (model 1) we express $\eta_{i,j}$ the linear predictor corresponding to $y_{i,j}$ as

$$\eta_{i,j} = \exp\left[\alpha + \beta \cdot \text{SEP}_{i,j} + \log(\text{exposure}_{i,j})\right], \tag{1}$$

where α is a global intercept, $\text{SEP}_{i,j}$ is the SEP decile (numerical value from 1 to 10), β is a regression coefficient (i.e., $\exp(\beta)$ is the incidence rate ratio for one unit increase of SEP decile), and $\log(\text{exposure}_{i,j})$ is the offset. We refer to this model as unadjusted with SEP as a continuous variable.

To assess whether including SEP as a continuous variable is adequate, we develop an alternative model (model 1') where

$$\eta_{i,j} = \exp\left[\alpha + \beta_2 \cdot \text{SEP-2}_{i,j} + \beta_3 \cdot \text{SEP-3}_{i,j} + \ldots + \beta_{10} \cdot \text{SEP-10}_{i,j} + \log(\text{exposure}_{i,j})\right], \tag{2}$$

where $\text{SEP-}k_{i,j}$ ($k \in \{2, \dots, 10\}$) is a dummy variable informing the count refers to individuals of SEP k. We compare this model to model 1 using model selection tools in order to assess whether the continuous assumption of model 1 is adequate. This continuous formulation is necessary to avoid inflating the number of parameters when introducing interactions (nine parameters are approximated with one).

The next model (model 2) introduces adjustment on age, sex, epidemic wave and an interaction between SEP and canton, with the linear predictor expressed as

$$\begin{aligned}\eta_{i,j} = \exp[&\alpha_j + \beta_j \cdot \text{SEP}_{i,j} + \gamma_1 \cdot \text{Age}_{2,i,j} + \ldots + \gamma_8 \cdot \text{Age}_{9,i,j} + \delta \cdot \text{Sex}_{i,j} + \\ &\zeta \cdot \text{Wave}_{i,j} + \iota \cdot \text{SEP}_{i,j} \cdot \text{Period}_{i,j} + \log(\text{exposure}_{i,j})].\end{aligned} \tag{3}$$

In this formulation, the intercept α_j can vary by canton (this type of formulation can be referred to as a "random intercept"). The regression coefficient for SEP β_j is also allowed to vary by canton ("random slope"). This implies that there are now 26 parameters α_j and 26 parameters β_j in the model, themselves controlled by hyperparameters:

$$\alpha_j = \mu_\alpha + \sigma_\alpha \cdot a_j \qquad \text{with } a_j \sim \mathcal{N}(0, 1) \tag{4}$$

$$\beta_j = \mu_\beta + \sigma_\beta \cdot b_j \qquad \text{with } b_j \sim \mathcal{N}(0, 1) \tag{5}$$

Note that because of the exponential link function, the estimated parameters a_j and b_j have a multiplicative effect. The remaining elements of equation are regression coefficients for the age groups (in 9 groups, from 0-9 to 80+), for the sex and for the epidemic wave (2 waves).

Following the same principles, we also develop a last model (model 3) that includes interactions between SEP and age group, SEP and sex and SEP and epidemic wave.

In all models, the likelihood can be expressed as

$$\Pr(\theta | y) = \prod_i \prod_j \text{negative-binomial}(y_{i,j} | \eta_{i,j}, \phi), \tag{6}$$

where ϕ is the overdispersion parameter and θ corresponds to the set of all parameters and hyperparameters to be estimated. The models are implemented in `R` using package `rstanarm` [1]. We select weakly-informative priors for all parameters, that is normal distributions with mean 0 and standard deviation 2.5 for intercept and slope parameters and exponential distributions with rate 1 for overdispersion and variance parameters [2]. All code is available at `https://github.com/jriou/covid-sep-ch`.

3 Extended results

3.1 Model selection

Model selection was based on the leave-one-out information criterion (LOOIC) [3]. This quantity evaluates the pointwise out-of-sample prediction accuracy from a fitted Bayesian model using the log-likelihood evaluated at the posterior simulations of the parameter values. LOOIC values can be compared across models fitted to the same dataset by computing the pointwise difference in predictive accuracy. As for other information criterions

such as the Akaike Information Criterion, a decrease in LOOIC corresponds to a better model fit. Rather than using a fixed threshold, LOOIC differences must be assessed with regards to their uncertainty, for instance using the standard error. A common approach is to consider a model as better than another if the LOOIC difference is negative with an absolute value greater than two times its standard error.

Table S2 present LOOIC estimates for models 1 to 3 and LOOIC differences with model 1 as a reference. We see that the difference between models 1 and 2 is quite small overall, and generally positive, meaning that model 1 generally has a better fit than model 1'. On the other hand, model 2 is clearly an improvement over model 1.

Table S2: Model selection based on left-one-out information criterion (LOOIC). Three models are compared: unadjusted model with SEP as a continuous variable (model 1), unadjusted model with SEP as a discrete variable (model 1') and adjusted model with SEP as a continuous variable (model 2, adjustment on age, sex, epidemic period and canton).

Outcome	Denominator	LOOIC (se) for model 1	LOOIC (se) for model 1'	Difference (se) with model 1	LOOIC (se) for model 2	Difference (se) with model 1
Total tests	Per population	104,775 (490)	104,783 (490)	+8 (3)	78,657 (494)	-26,118 (216)
Positive tests	Per population	77,890 (397)	77,901 (397)	+11 (3)	59,149 (392)	-18,740 (188)
Positive tests	Per test	48,610 (402)	48,597 (402)	-12 (11)	37,563 (405)	-11,046 (184)
Hospitalisations	Per population	32,391 (327)	32,406 (328)	+14 (5)	21,654 (242)	-10,737 (177)
Hospitalisations	Per test	21,770 (309)	21,782 (309)	+12 (4)	14,216 (226)	-7,555 (142)
Hospitalisations	Per positive test	29,166 (264)	29,175 (264)	+9 (5)	20,589 (217)	-8,577 (144)
ICU admissions	Per population	10,080 (210)	10,088 (210)	+9 (5)	6,844 (152)	-3,236 (111)
ICU admissions	Per test	7,015 (179)	7,023 (179)	+8 (6)	4,598 (130)	-2,418 (93)
ICU admissions	Per positive test	9,594 (197)	9,603 (197)	+10 (5)	6,626 (146)	-2,967 (103)
Deaths	Per population	16,486 (322)	16,498 (322)	+12 (4)	9,559 (210)	-6,928 (157)
Deaths	Per test	11,121 (269)	11,132 (269)	+11 (3)	6,141 (172)	-4,980 (135)
Deaths	Per positive test	14,952 (273)	14,965 (273)	+13 (2)	8,176 (173)	-6,775 (149)

3.2 Model fit

Figure S3 shows the posterior predictive check of the adjusted model (model 2) applied to all relevant combinations of outcome and denominator. The fit is generally very good, with most data point falling into the 95% prediction intervals.

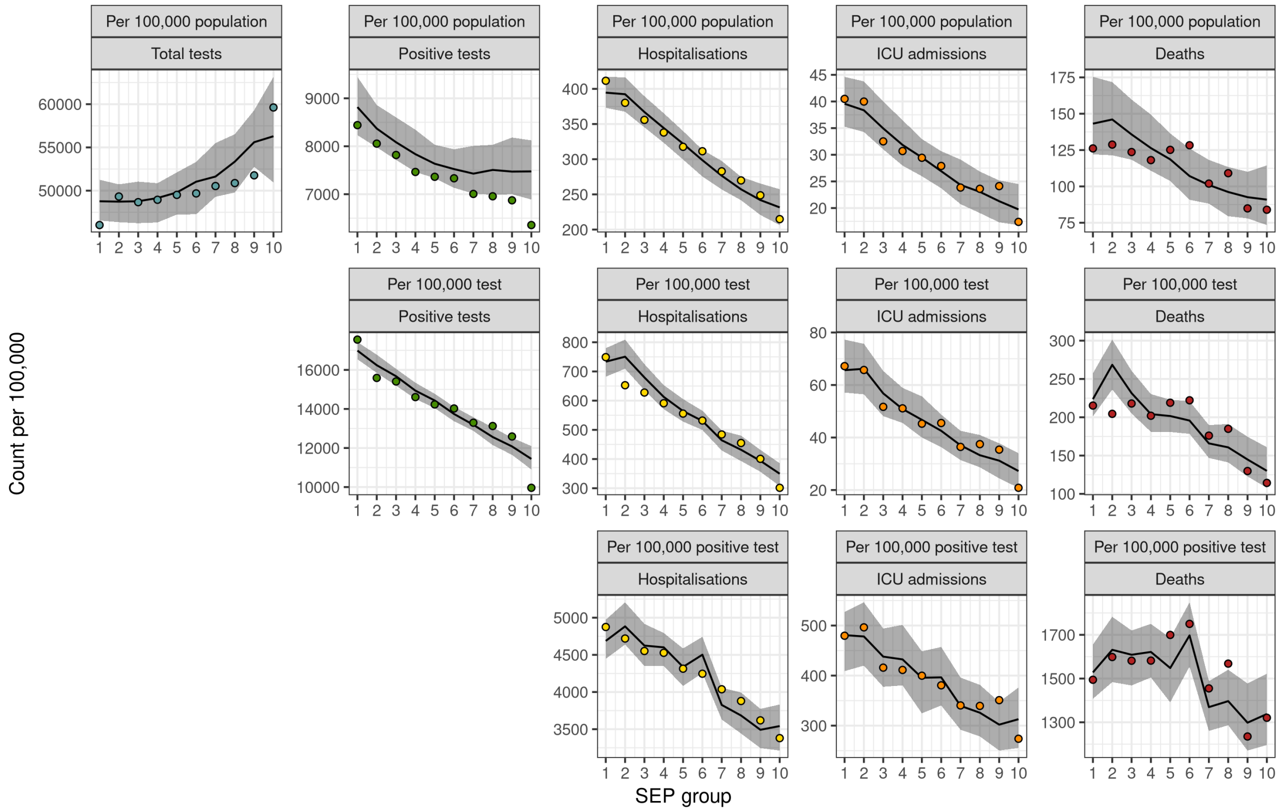

Figure S3: Fit of the models adjusted for age, sex, epidemic wave and canton. Circles show data points, the line and shaded area show the corresponding model prediction (median and 95% prediction interval).

We spot potential issues in three areas. First, the number of total tests per 100,000 population is not well-predicted by the model in the highest SEP decile, suggesting that the continuous formulation could be inadequate. Still, LOOIC comparisons between model 1 (continuous) and model 1' (discrete) in Table S2 don't suggest an advantage in using a discrete formulation. Moreover, a discrete formulation would lead to huge inflation of the number of parameters when including interactions. We decide to continue with the continuous formulation and consider testing in the 10th decile as an outlier.

Second, the number of positive tests per 100,000 tests is also not well-predicted in the highest SEP decile. This issue mirrors the previous one, but this time there is a weak indication that the discrete formulation might fit better (LOOIC difference -12 with a standard error of 11). Still, considering the difficulty posed by using 9 parameters instead of 1 for the effect of SEP, we go ahead with the continuous approach.

Third, the number of positive tests per 100,000 population is not well-described for the SEP deciles 7, 8, 9 and 10. This issue is not related to the continuous formulation, as the comparison between models 1 and 2 shows. Rather, this discrepancy comes from an interaction between the effect of SEP and the epidemic wave, as detailed in section 4.1.

3.3 Socio-economic position

Table S3 presents additional results about models 1, 1' and 2. We present incidence rate ratios (IRRs) per SEP decile for models 1, corresponding to $\exp(\beta)$ in equation 1. For model 2, the IRRs per SEP decile correspond to the average effect of one SEP decile across cantons, $\exp(\mu_\beta)$ in equation 5. In the models using a continuous formulation (1 and 2), IRRs for the 10th decile of SEP compared to 1st can be computed from the IRRs per SEP decile to the power of 9. For the model with a discrete formulation (model 1'), the IRRs for the 10th decile of SEP compared to 1st correspond to $\exp(\beta_{10})$ in equation 2. For this model, we present the IRRs for each group of SEP (compared to the first) in Figure S4.

IRRs per SEP decile are all similar between models 1 and 2, suggesting that while the adjustment on age, sex, epidemic wave and canton clearly improved the model fit (see Table S2), the association between SEP and each outcome remain consistent. Comparing the IRRs for the 10th decile of SEP compared to the 1st allows comparing all three models, and again assess the adequacy of the continuous formulation. The estimates are mostly in agreement, comforting our approach. We observe a difference in the total tests per population and in positive tests per tests, corresponding to outliers in the 10th SEP decile as discussed above.

Table S3: Association of deciles of neighbourhood index of socioeconomic position (SEP) with five outcomes related to SARS-CoV-2 surveillance and care: total tests, positive tests, hospitalisations, intensive care unit (ICU) admissions and deaths. Three denominators are considered accordingly: population, total tests and positive tests. Three models are considered: unadjusted model with SEP as a continuous variable (model 1), unadjusted model with SEP as a discrete variable (model 1') and adjusted model with SEP as a continuous variable (model 2, adjustment on age, sex, epidemic period and canton).

Outcome	Denominator	IRR per SEP decile for model 1	IRR for 10th decile compared to 1st for model 1	IRR for 10th decile compared to 1st for model 1'	IRR per SEP decile for model 2	IRR for 10th decile compared to 1st for model 2
Total tests	Per population	1.02 (1.01-1.04)	1.21 (1.07-1.36)	1.34 (1.14-1.58)	1.02 (1.00-1.03)	1.18 (1.02-1.36)
Positive tests	Per population	0.99 (0.98-1.00)	0.90 (0.81-0.99)	0.88 (0.76-1.02)	0.99 (0.98-1.01)	0.95 (0.84-1.11)
Positive tests	Per test	0.97 (0.97-0.98)	0.76 (0.73-0.80)	0.68 (0.63-0.73)	0.97 (0.96-0.98)	0.75 (0.69-0.81)
Hospitalisations	Per population	0.94 (0.92-0.95)	0.57 (0.49-0.65)	0.50 (0.39-0.62)	0.94 (0.92-0.96)	0.56 (0.49-0.67)
Hospitalisations	Per test	0.94 (0.92-0.95)	0.56 (0.48-0.66)	0.48 (0.37-0.61)	0.92 (0.91-0.94)	0.49 (0.43-0.56)
Hospitalisations	Per positive test	0.96 (0.95-0.98)	0.72 (0.63-0.83)	0.62 (0.50-0.77)	0.96 (0.95-0.97)	0.68 (0.62-0.74)
ICU admissions	Per population	0.92 (0.89-0.94)	0.45 (0.36-0.57)	0.40 (0.28-0.57)	0.90 (0.87-0.93)	0.39 (0.28-0.53)
ICU admissions	Per test	0.90 (0.87-0.93)	0.39 (0.29-0.52)	0.29 (0.18-0.45)	0.90 (0.88-0.93)	0.40 (0.31-0.53)
ICU admissions	Per positive test	0.93 (0.91-0.96)	0.53 (0.41-0.68)	0.44 (0.30-0.65)	0.93 (0.91-0.96)	0.54 (0.43-0.70)
Deaths	Per population	0.96 (0.93-0.99)	0.71 (0.54-0.94)	0.78 (0.51-1.21)	0.96 (0.92-1.01)	0.71 (0.49-1.10)
Deaths	Per test	0.98 (0.94-1.01)	0.80 (0.58-1.09)	0.78 (0.50-1.24)	0.96 (0.94-0.98)	0.66 (0.57-0.82)
Deaths	Per positive test	0.97 (0.95-1.00)	0.79 (0.62-1.03)	0.80 (0.55-1.18)	0.98 (0.97-1.00)	0.86 (0.76-0.99)

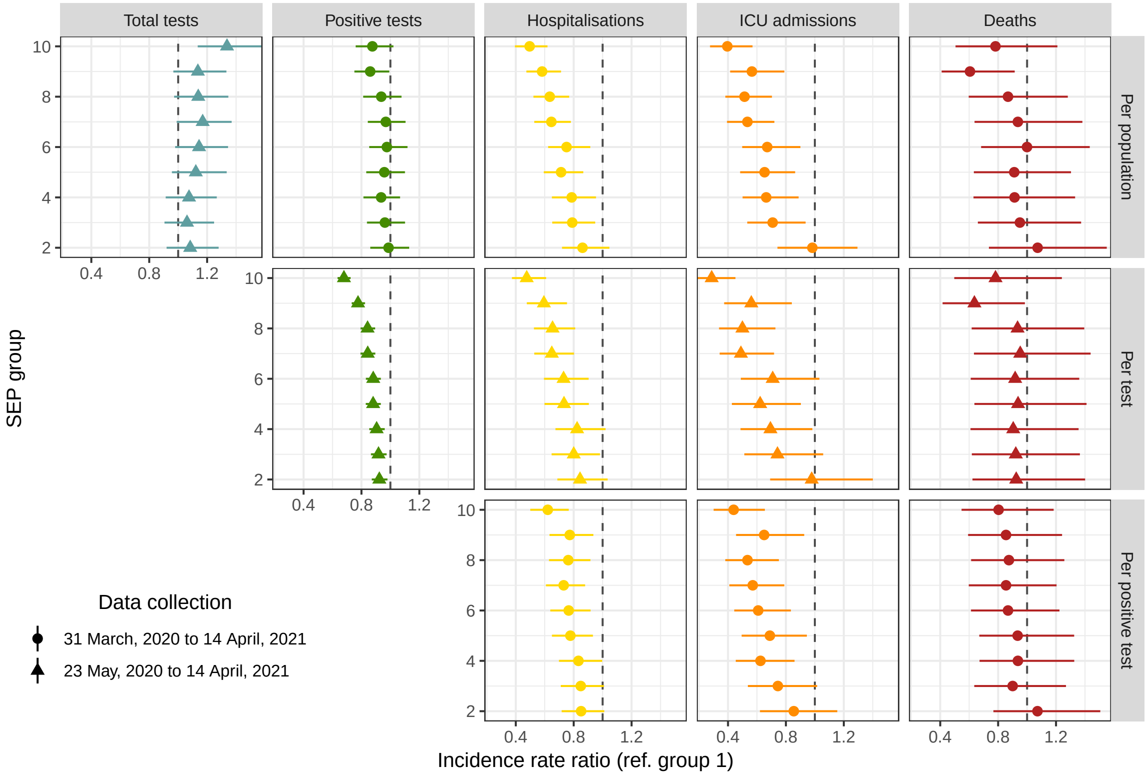

Figure S4: Unadjusted incidence rate ratios (IRR) for each group of neighbourhood socioeconomic position (SEP) compared to the first group (model 1') for the counts of SARS-CoV-2 tests, positive tests, hospitalisations, ICU admissions and mortality per population, tests or positive tests. Median posteriors and 95% credible interval are shown in each case.

3.4 Other covariates

Because of their known or suspected role on SARS-CoV-2 infection natural history and care, estimates of the association between SEP and each outcome was adjusted for age, sex, epidemic wave and canton.

Figure S5 presents an overview of the association between age, sex and epidemic wave and each outcome. The association is presented as incidence rate ratios, and must be interpreted relatively to a reference group (identified with squares). For instance, we observe that there has been almost 100 times more total tests per population during the second wave than during the first wave. These results all come from the same models that includes age, sex, epidemic wave, canton and SEP decile.

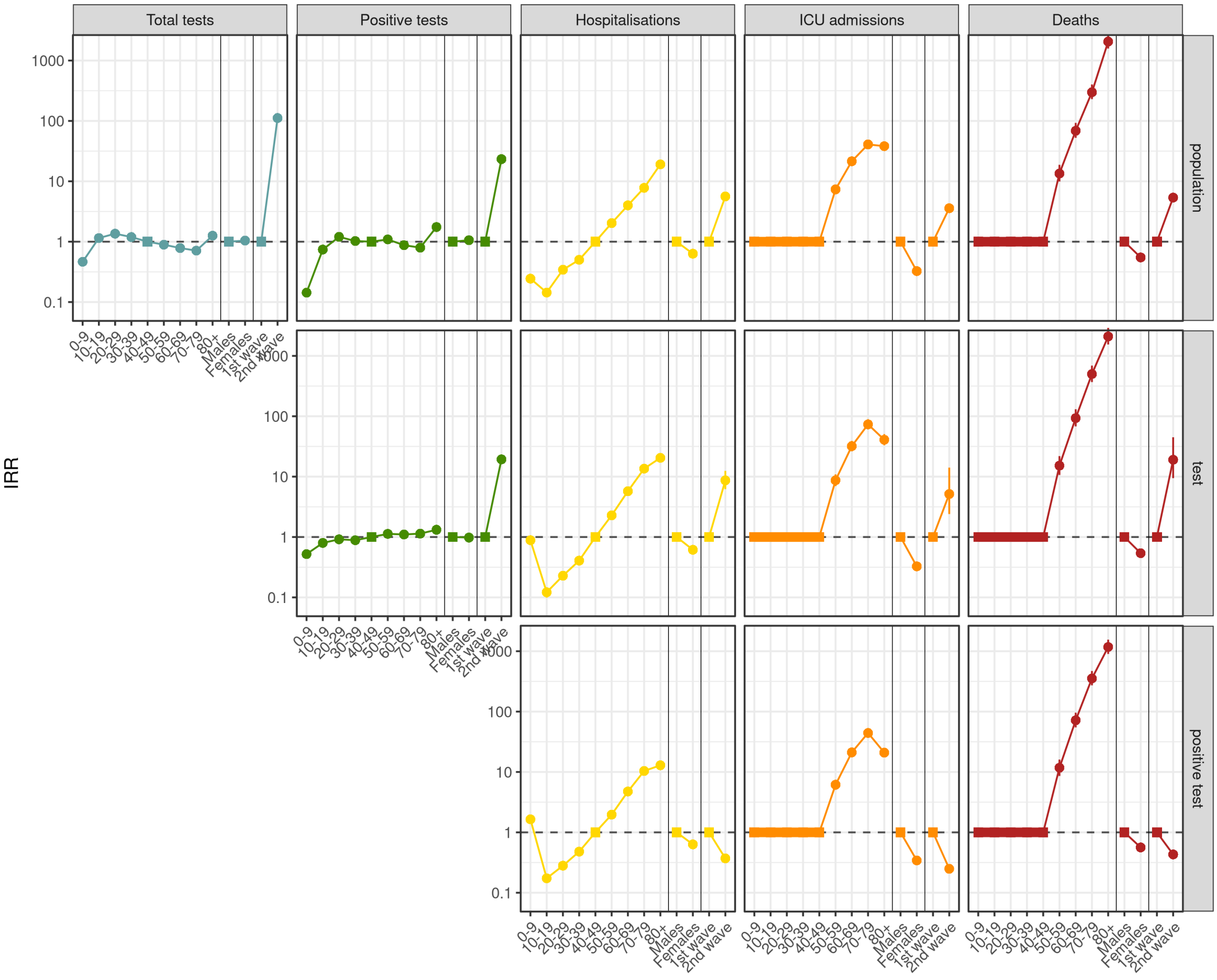

Figure S5: Incidence rate ratios (95% credible interval) for the association between age, sex and epidemic wave and five outcomes related to SARS-CoV-2 surveillance and care: total tests, positive tests, hospitalisations, intensive care unit (ICU) admissions and deaths. The reference group for age is 40-49 (squares) for total tests, positive tests and hospitalisations. Because of the small number of events in younger groups, we pooled together age groups 0 to 49 for ICU admissions and deaths, and used this larger group as reference. The reference group is males and the first epidemic wave. The models are also adjusted for canton and SEP decile.

Figure S6 shows the association between canton and each outcome. As the canton effect was formulated using a random intercept, the interpretation does not rely upon a reference group but to the overall average. For instance, there has been around 3 times more deaths per population in canton Ticino (TI) compared to average. The geographic heterogeneity can be assessed by comparing the spread of cantons, or by looking at the inter-cantonal variance (Figure S7). This inter-cantonal variance corresponds to σ^2_α in equation 5. For instance, there is more inter-cantonal variation in the number of deaths per population (more affected by the total size of the epidemic) than in the number of deaths per positive test (more affected by the quality of care).

Tables S6, S7 and S8 present the same estimates in tabular form.

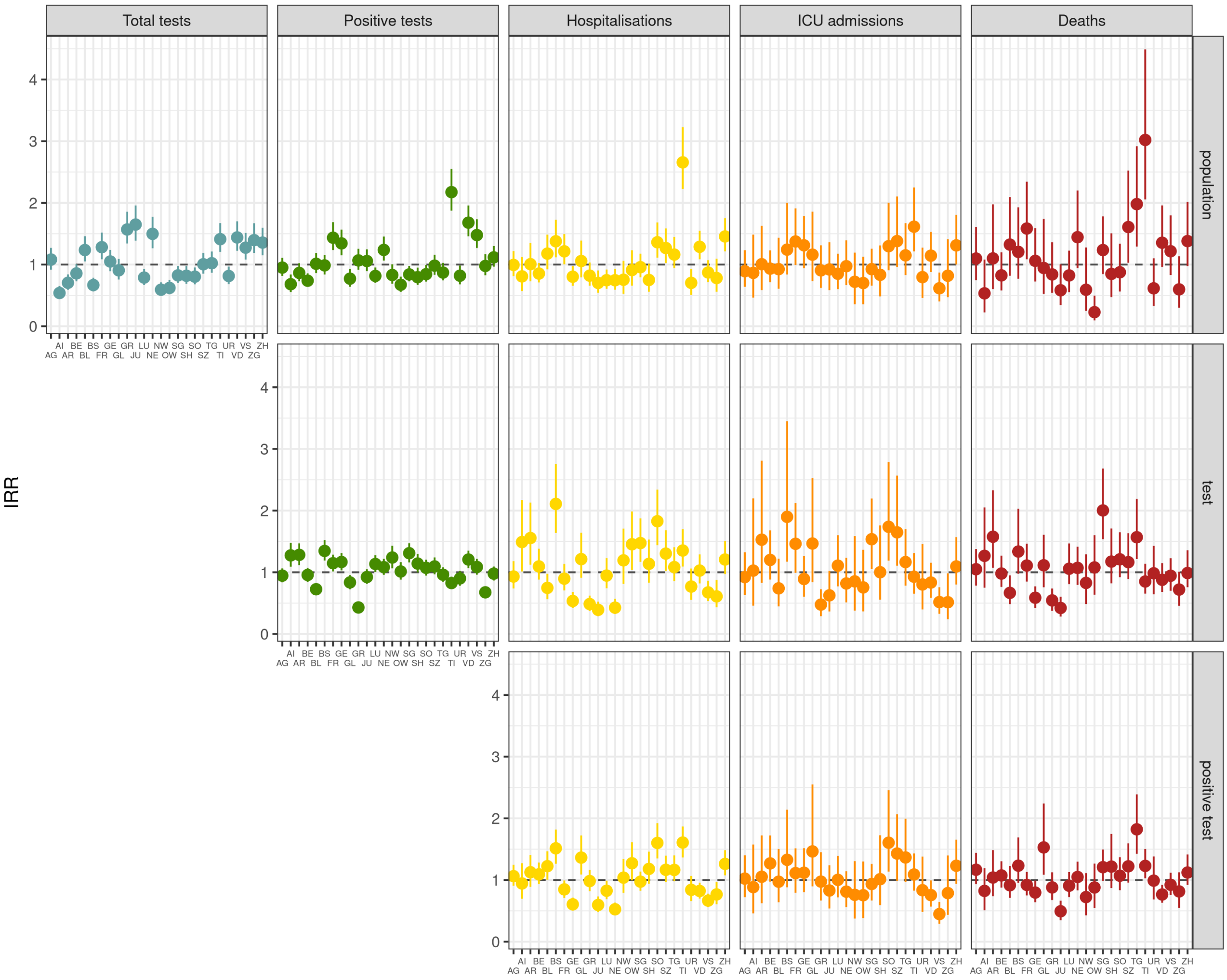

Figure S6: Incidence rate ratios (95% credible interval) for the association between each canton and five outcomes related to SARS-CoV-2 surveillance and care: total tests, positive tests, hospitalisations, intensive care unit (ICU) admissions and deaths. As this is implemented as a random intercept, there is no reference groups and the incidence rate ratios must be interpreted relatively to the overall average. The models are also adjusted for age, sex, epidemic wave and SEP decile.

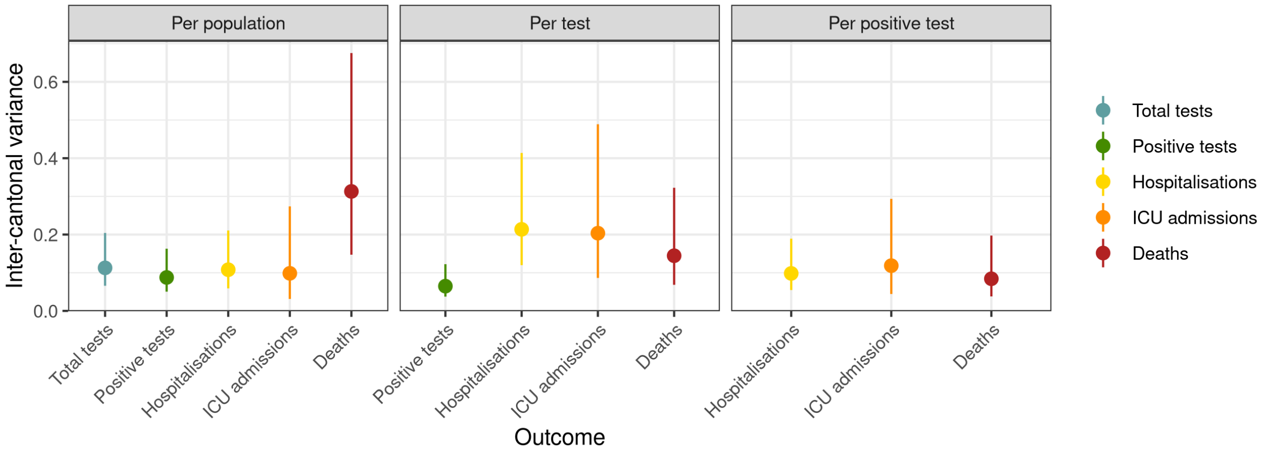

Figure S7: Inter-cantonal variance (95% credible interval) of the association between each canton and five outcomes related to SARS-CoV-2 surveillance and care: total tests, positive tests, hospitalisations, intensive care unit (ICU) admissions and deaths.

3.5 Interaction analysis

We now consider two-way interactions between each covariate (age, sex, epidemic wave and canton) and SEP, to assess whether the association between SEP and each outcome is modified by these other factors. We start with the canton, as the interaction with canton is already included in model 2 (the adjusted model that is used for the main results) as a "random slope" (see equations 4 and 5).

Figure S8 shows estimates of the association between SEP and each outcome (IRR per SEP decile) in each canton. Going back to equation 5, these correspond to $\exp(\beta_j)$ for each canton j, computed from the average effect of SEP μ_β, the inter-cantonal variance σ^2_β and from the deviation in each canton (the random slope) b_j. We observe large deviations from average in some cantons, for instance in Geneva (GE) for total tests per population. This can be interpreted as: the positive association between SEP and total tests per population is even steeper in Geneva compared to average. More generally, we observe a reduction in cantonal heterogeneity as we move from the population denominator to the test or positive test. This can be quantified by the estimate of inter-cantonal variance (Figure S9).

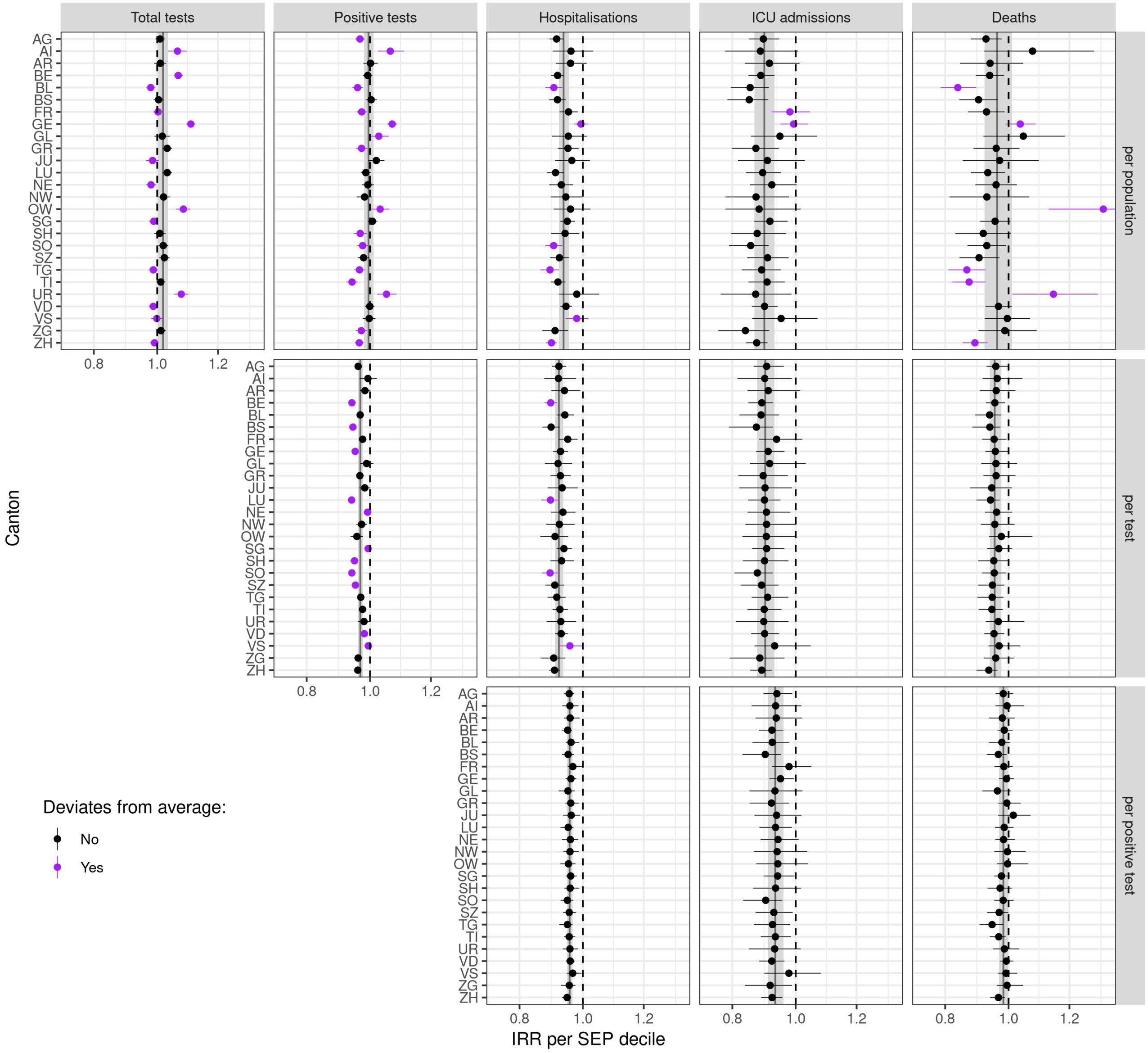

Figure S8: Incidence rate ratios (95% credible interval) for the association between socio-economic position (SEP) and five outcomes, *separately in each canton*. Deviation from average (highlighted in purple) is defined as the 95% credible interval in the canton not including the overall average (shown by the full line and shaded area). Credible intervals are truncated at 1.3 for visibility, exact numbers are available in Table S10.

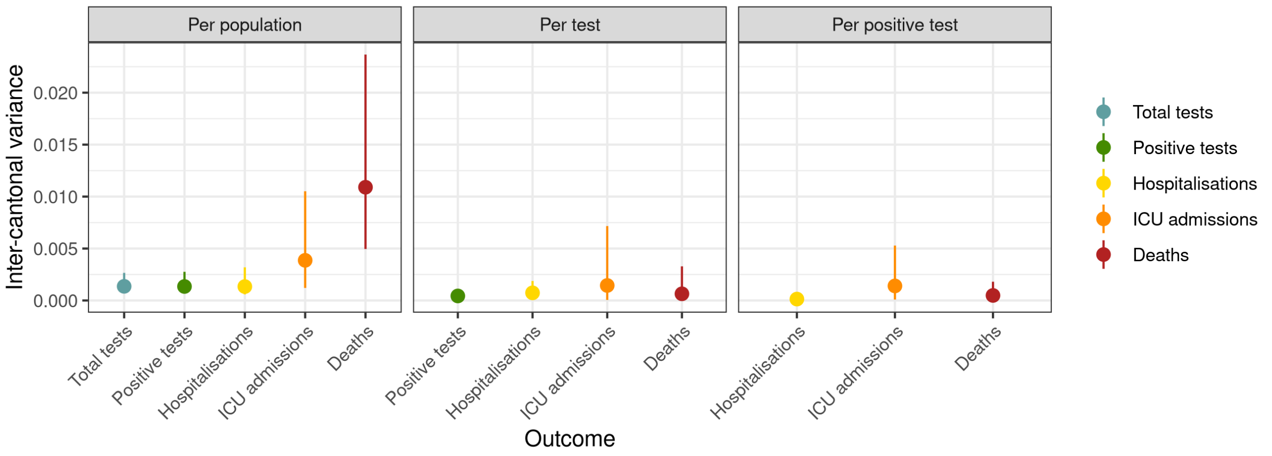

Figure S9: Inter-cantonal variance (95% credible interval) of the association between SEP and five outcomes related to SARS-CoV-2 surveillance and care, *separately in each canton.*

Figure S10 shows estimates of the association between SEP and each outcome (IRR per SEP decile) by age, sex and epidemic wave. These estimates come from model 3, including all two-way interactions. We observe for all combinations of outcome and denominator that the strength of association between SEP and the outcome decreases in older age groups. This observation is especially meaningful for deaths per population, per test and per positive tests, because a large proportion of deaths (71%) occur in the 80+ age group, where the association between SEP and mortality is the weakest (IRR close to 1). The fact that the association between SEP and mortality appears strongly in other age groups (IRRs consistently below 1) suggests that the absence of association found on average in the population is misleading, and that SEP and mortality are actually associated in most age groups. We don't find evidence that the association between SEP and each outcomes varies systematically according to sex or epidemic period. One exception is positive tests per population, where we observe a diminution of the IRR between the first and second epidemic wave. This point is further developed in section 4.1. Estimates obtained with tests as a denominator show large uncertainty during the first wave, because total tests were only available from 23 May 2020 onwards, meaning that the estimate for the first wave (before 8 June) is based on only 2 weeks of data. These should be interpreted with caution.

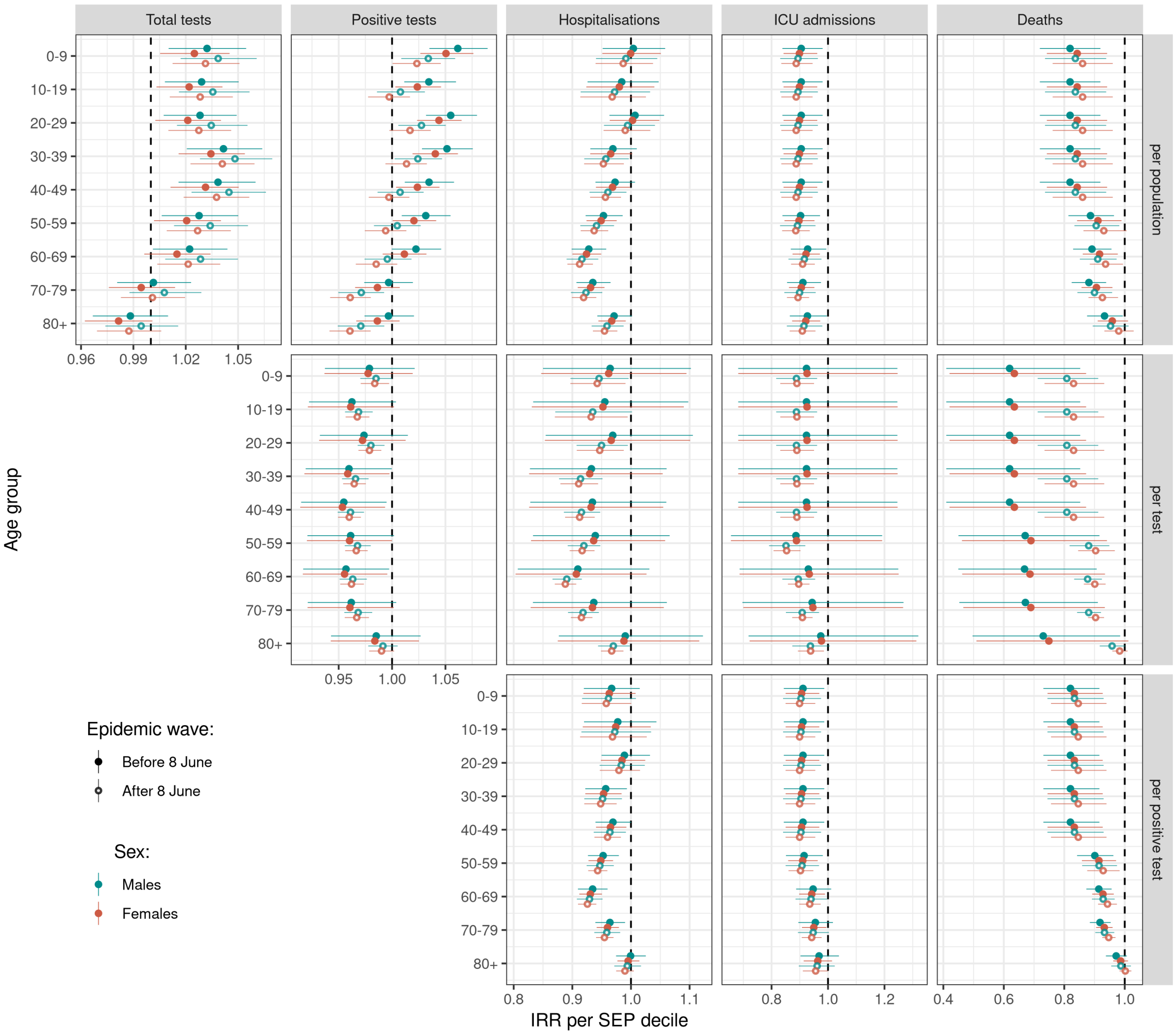

Figure S10: Incidence rate ratios (95% credible interval) for the association between socio-economic position (SEP) and five outcomes, *separately in each combination of age group, sex and epidemic wave.*

3.6 Time delays between key events

Available data lacks direct information about disease severity. A measure of disease severity among hospitalised individuals would be required to assess whether individuals with higher SEP make a disproportionate use of hospital resources. An indirect approach is to examine the delays between key events such as symptom onset and hospitalisation date. Figure S11 shows the mean delays between key events (symptom onset, sampling date, test date, hospitalisation date and death date), stratified by SEP group. We don't find any clear trend that could be suggesting a difference in disease severity.

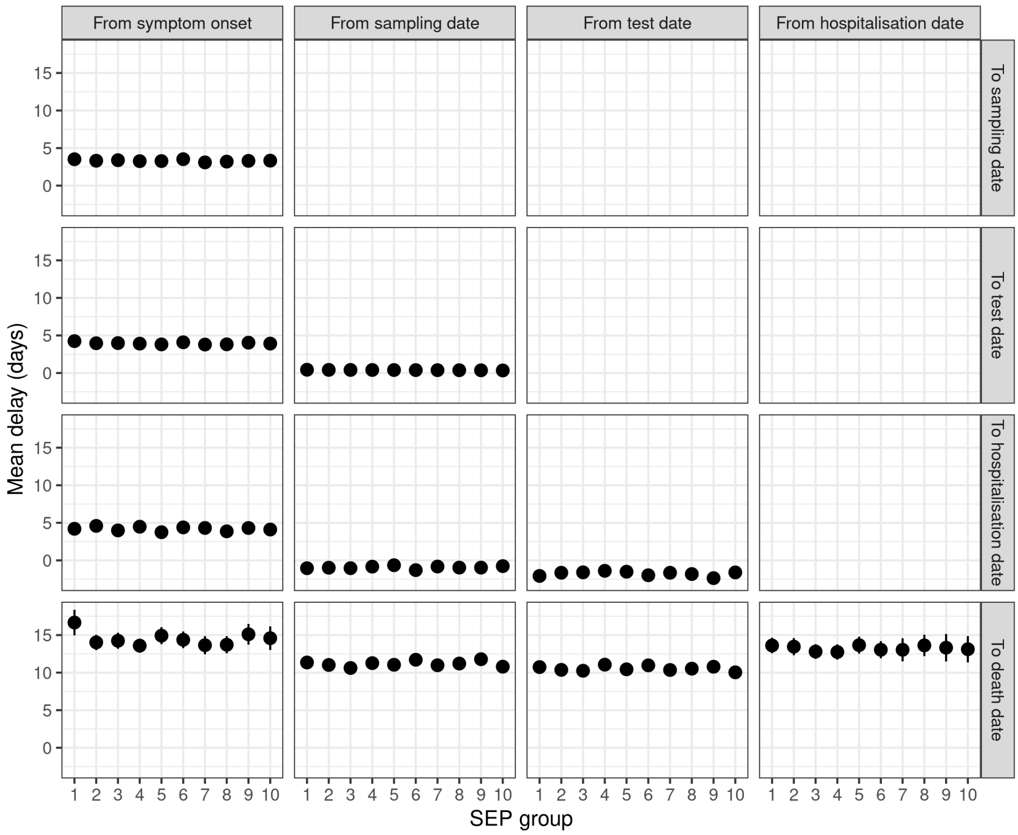

Figure S11: Mean delays in days (with 95% confidence intervals) between key events: symptom onset, sampling date, test date, hospitalisation date and death date, stratified by SEP group.

4 Sensitivity analyses

4.1 Stratification by period

The issue with model fit for positive tests per population explained in section 3.2, together with the interaction between SEP and epidemic wave spotted in section 3.5 led us to further explore the effect of epidemic wave on the results. To do that, we repeated the analyses after stratifying by epidemic wave (before/after 8 June, 2020). Figure S12 shows the model fit after applying the stratification. We observe a clear improvement of the model fit to positive tests per population (i.e., test positivity) and deduce the cause of the issue. The association between SEP and test positivity changed from being positive (IRR per SEP decile: 1.02 [95%CrI: 1.01-1.03]) during the first wave to slightly negative (IRR per SEP decile: 0.99 [95%CrI: 0.98-0.99]) during the second wave. In accordance with the results from the interaction analyses, we didn't find other differences between epidemic waves (Figure S13). We also don't see clear differences in the effect of other covariates (Figure S14).

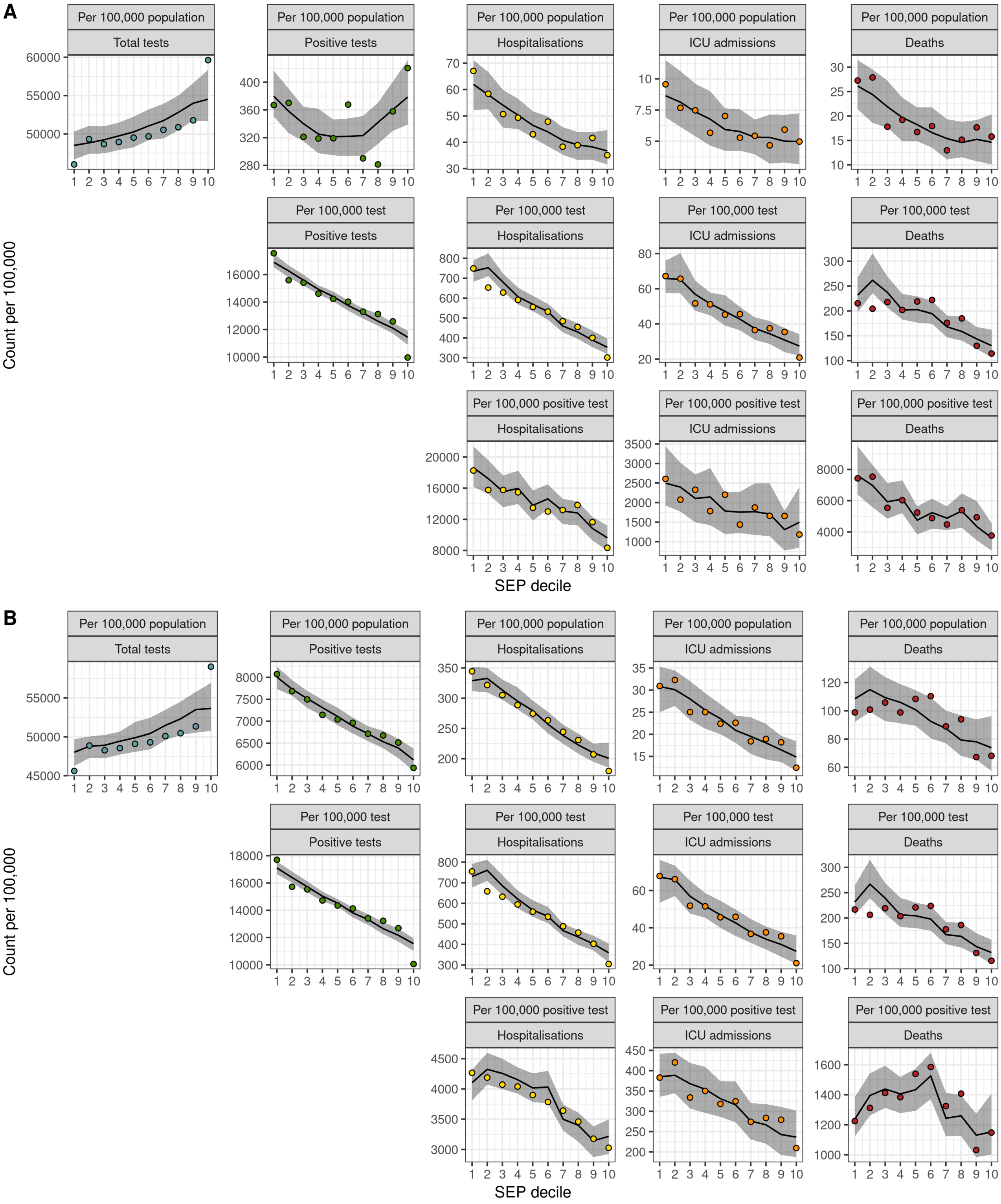

Figure S12: Fit of the models adjusted for age, sex, epidemic wave and canton by epidemic wave (panel A: 1st wave until 8 June, 2020; panel B: 2nd wave from 8 June, 2020). Circles show data points, the line and shaded area show the corresponding model prediction (median and 95% prediction interval).

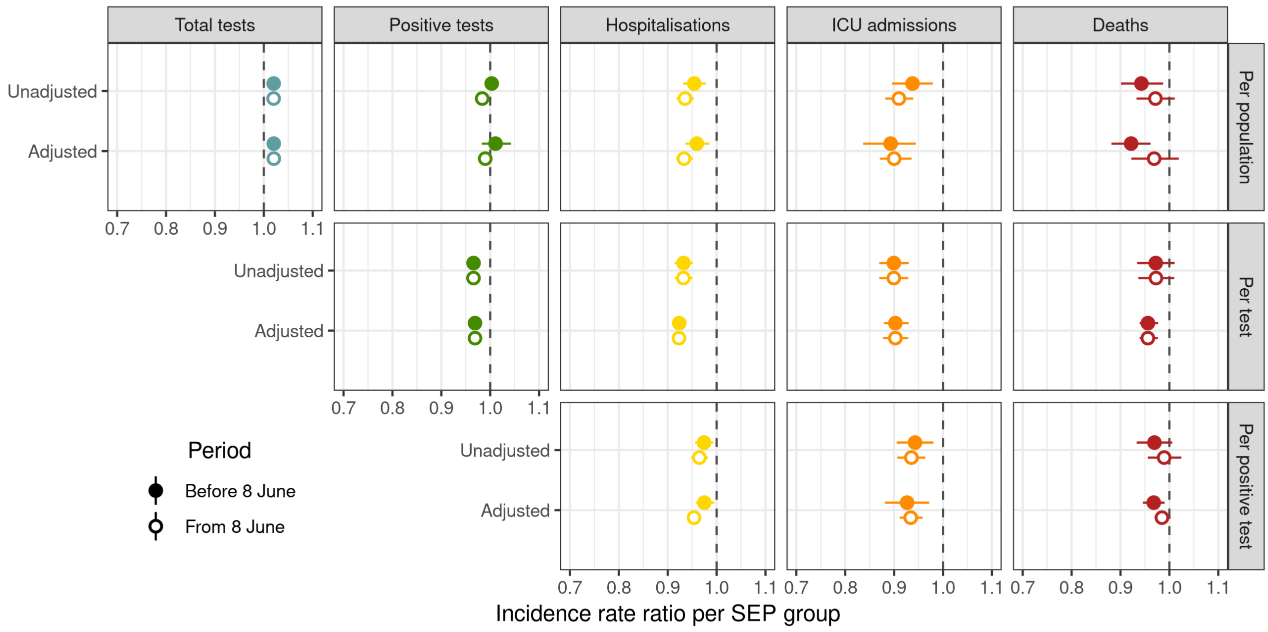

Figure S13: Estimates of incidence rate ratios per decile of SEP stratified by epidemic wave.

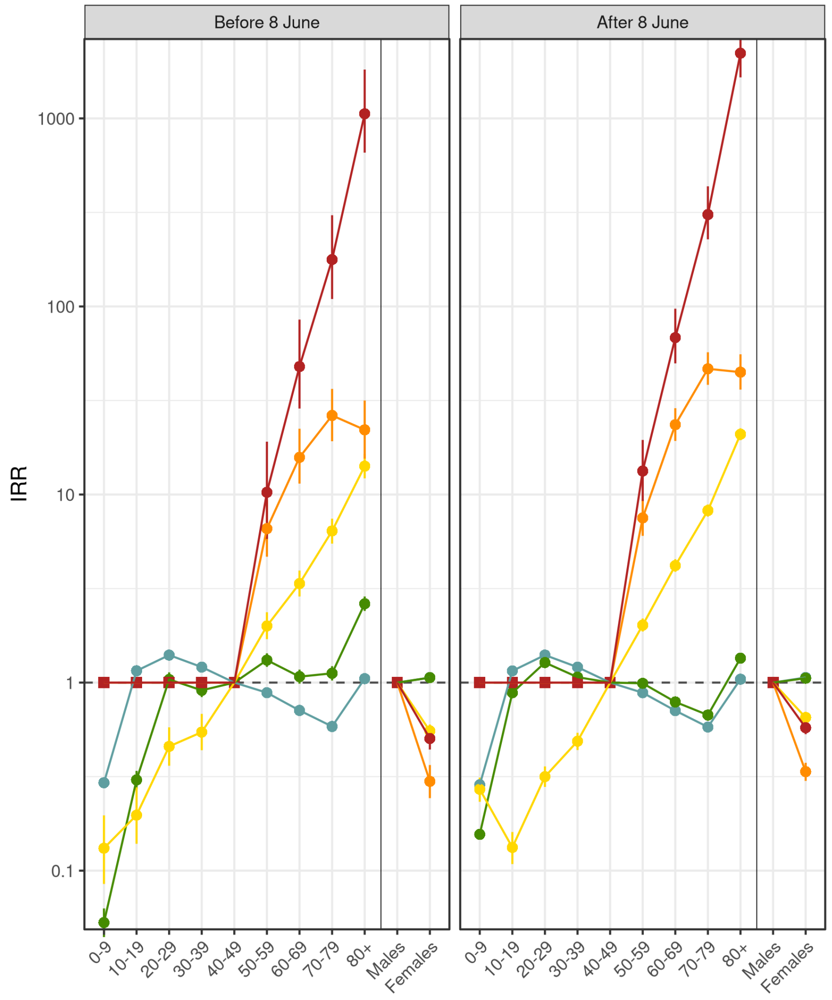

Figure S14: Estimates of incidence rate ratios between age and sex stratified by epidemic wave.

4.2 Excluding geocodes based only on ZIP code

A small proportion of individuals did not provide a full residential address but only a ZIP codes (Table S1). In these situations, the attributed geocode corresponded to the centroïd of the ZIP code area. As ZIP codes might show large heterogeneity with regards to the SEP, this may lead to misclassification and influence the results. We repeated the analysis removing these cases from the dataset, and obtained very similar results (Figure S15).

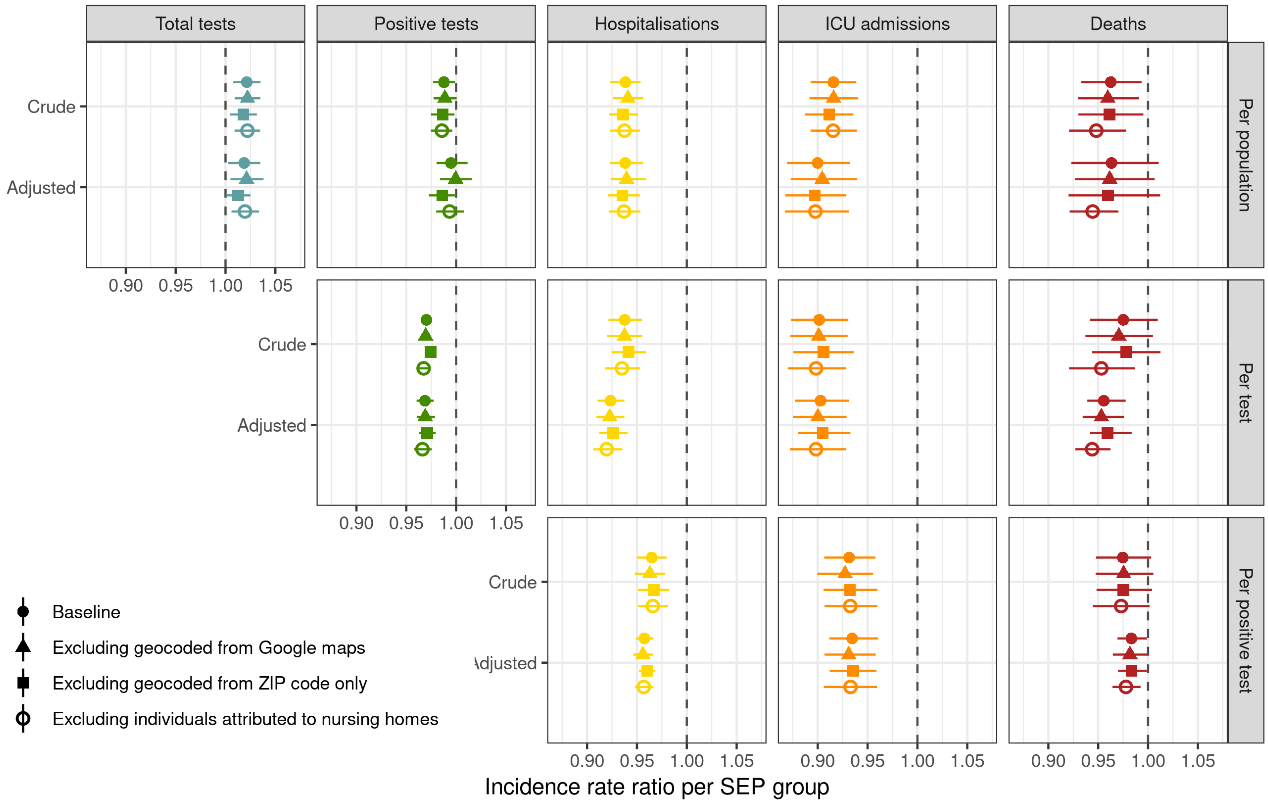

Figure S15: Estimates of incidence rate ratios per decile of SEP in the baseline analysis and in two sensitivity analyses: excluding geocodes based only on ZIP codes only, and excluding geocodes attributed to retirement or nursing homes.

4.3 Excluding geocodes obtained with Google maps

Whenever the official Swisstopo geocoding software failed to obtain a geocode from the residential address, geocoding was attempted using the Google maps API (with specific precautions as explained in the main text). Google maps may lead to better results in case of typos or mistakes in the residential address [4]. We conducted a sensitivity analysis excluding geocodes obtained with the Google maps API, and obtained very similar results (Figure S15).

4.4 Excluding geocodes attributed to retirement or nursing homes

With our geocoding procedure, residents of retirement or nursing homes are attributed to a SEP decile corresponding to the area of the retirement or nursing home, and not the area where they lived before . This may lead to misclassification and influence the results, especially regarding mortality as they may represent a large proportion of deaths. Having no direct way of identifying residents of retirements or nursing homes, we considered a sensitivity analysis whereby individuals with addresses geocoded within 25 meters of a retirement or nursing home was excluded (Table S1). With this assumption, we identified 3,178 of the COVID-19-related deaths (33.9%). This proportion increased from 16.4% (258/1,575) during the first wave to 37.4% (2,920/7,808) during the second wave. We also checked the impact of changing the threshold to 50 meters instead of 25 meters. This increased the number of deaths attributed to residents of retirement or nursing homes to 3,489 (37.2%).

We repeated the analysis removing these cases from the dataset (with the 25 meters threshold). The results regarding the association between SEP and tests, positive tests, hospitalisations and ICU admissions were very similar (Figure S15). Interestingly, removing residents of retirement or nursing homes changed the association

between SEP and deaths per population, deaths per test and deaths per positive test. In the baseline analysis the association between SEP and mortality was unclear, with a 95% credible interval including 1 for deaths per population and deaths per positive test. Removing these individuals led to a clear negative association with all three denominators, with 95% credible intervals excluding 1 in all cases (Table S4 and S5). This might be linked to both potential misclassification and the effect of age.

Table S4: Estimates of incidence rate ratios per decile of SEP in the baseline analysis and in two sensitivity analyses: excluding geocodes based only on ZIP codes only, and excluding geocodes attributed to retirement or nursing homes.

Outcome	Denominator	Baseline	Excluding geocoded from ZIP code only	Excluding geocoded from Google maps	Excluding individuals attributed to nursing homes
Total tests	Per population	1.02 (1.00-1.03)	1.01 (1.00-1.03)	1.02 (1.00-1.04)	1.02 (1.01-1.03)
Positive tests	Per population	0.99 (0.98-1.01)	0.99 (0.97-1.00)	1.00 (0.98-1.02)	0.99 (0.98-1.01)
Positive tests	Per test	0.97 (0.96-0.98)	0.97 (0.96-0.98)	0.97 (0.96-0.98)	0.97 (0.96-0.98)
Hospitalisations	Per population	0.94 (0.92-0.96)	0.94 (0.92-0.95)	0.94 (0.92-0.96)	0.94 (0.92-0.95)
Hospitalisations	Per test	0.92 (0.91-0.94)	0.93 (0.91-0.94)	0.92 (0.91-0.94)	0.92 (0.91-0.94)
Hospitalisations	Per positive test	0.96 (0.95-0.97)	0.96 (0.95-0.97)	0.96 (0.95-0.97)	0.96 (0.95-0.97)
ICU admissions	Per population	0.90 (0.87-0.93)	0.90 (0.87-0.93)	0.90 (0.87-0.94)	0.90 (0.87-0.93)
ICU admissions	Per test	0.90 (0.88-0.93)	0.91 (0.88-0.93)	0.90 (0.88-0.93)	0.90 (0.87-0.93)
ICU admissions	Per positive test	0.93 (0.91-0.96)	0.94 (0.91-0.96)	0.93 (0.91-0.96)	0.93 (0.91-0.96)
Deaths	Per population	0.96 (0.92-1.01)	0.96 (0.92-1.01)	0.96 (0.93-1.01)	0.94 (0.92-0.97)
Deaths	Per test	0.96 (0.94-0.98)	0.96 (0.94-0.98)	0.95 (0.93-0.98)	0.94 (0.93-0.96)
Deaths	Per positive test	0.98 (0.97-1.00)	0.98 (0.97-1.00)	0.98 (0.96-1.00)	0.98 (0.96-0.99)

Table S5: Estimates of incidence rate ratios for the 10th decile of SEP compared to the 1st decile in the baseline analysis and in two sensitivity analyses: excluding geocodes based only on ZIP codes only, and excluding geocodes attributed to retirement or nursing homes.

Outcome	Denominator	Baseline	Excluding geocoded from ZIP code only	Excluding geocoded from Google maps	Excluding individuals attributed to nursing homes
Total tests	Per population	1.18 (1.02-1.36)	1.12 (1.01-1.25)	1.21 (1.05-1.40)	1.19 (1.06-1.35)
Positive tests	Per population	0.95 (0.84-1.11)	0.88 (0.78-1.00)	1.00 (0.86-1.15)	0.94 (0.83-1.07)
Positive tests	Per test	0.75 (0.69-0.81)	0.77 (0.71-0.83)	0.75 (0.70-0.82)	0.73 (0.68-0.80)
Hospitalisations	Per population	0.56 (0.49-0.67)	0.55 (0.48-0.65)	0.57 (0.49-0.69)	0.56 (0.48-0.65)
Hospitalisations	Per test	0.49 (0.43-0.56)	0.50 (0.44-0.58)	0.48 (0.42-0.56)	0.47 (0.41-0.55)
Hospitalisations	Per positive test	0.68 (0.62-0.74)	0.69 (0.64-0.75)	0.67 (0.61-0.74)	0.67 (0.62-0.74)
ICU admissions	Per population	0.39 (0.28-0.53)	0.38 (0.28-0.51)	0.41 (0.29-0.57)	0.38 (0.28-0.53)
ICU admissions	Per test	0.40 (0.31-0.53)	0.41 (0.32-0.53)	0.39 (0.30-0.51)	0.38 (0.29-0.51)
ICU admissions	Per positive test	0.54 (0.43-0.70)	0.55 (0.44-0.69)	0.53 (0.41-0.68)	0.54 (0.41-0.69)
Deaths	Per population	0.71 (0.49-1.10)	0.69 (0.47-1.11)	0.70 (0.50-1.06)	0.60 (0.48-0.76)
Deaths	Per test	0.66 (0.57-0.82)	0.69 (0.58-0.86)	0.65 (0.54-0.80)	0.60 (0.51-0.71)
Deaths	Per positive test	0.86 (0.76-0.99)	0.86 (0.76-0.99)	0.85 (0.72-1.01)	0.82 (0.72-0.93)

5 Tables of estimates

5.1 Other covariates

Table S6: Incidence rate ratios (95% credible interval) for the association between age and five outcomes related to SARS-CoV-2 surveillance and care (corresponding to Figure S5). The reference group is 40-49 for total tests, positive tests and hospitalisations. Because of the small number of events in younger groups, we pooled together age groups 0 to 49 for ICU admissions and deaths, and used this larger group as reference. The models are also adjusted for sex, epidemic wave, canton and SEP decile.

Outcome	Denominator	Age group	IRR
Total tests	population	0-9	0.46 (0.45-0.48)
Total tests	population	10-19	1.15 (1.10-1.19)
Total tests	population	0-29	1.36 (1.31-1.41)
Total tests	population	30-39	1.19 (1.15-1.24)
Total tests	population	50-59	0.89 (0.86-0.92)
Total tests	population	60-69	0.78 (0.75-0.82)
Total tests	population	70-79	0.71 (0.68-0.74)
Total tests	population	80+	1.26 (1.21-1.31)
Positive tests	population	0-9	0.14 (0.14-0.15)
Positive tests	population	10-19	0.74 (0.71-0.77)
Positive tests	population	0-29	1.20 (1.15-1.26)
Positive tests	population	30-39	1.02 (0.98-1.07)
Positive tests	population	50-59	1.09 (1.04-1.14)
Positive tests	population	60-69	0.87 (0.84-0.91)
Positive tests	population	70-79	0.80 (0.76-0.84)
Positive tests	population	80+	1.75 (1.67-1.83)
Positive tests	test	0-9	0.52 (0.50-0.54)
Positive tests	test	10-19	0.80 (0.78-0.82)
Positive tests	test	0-29	0.92 (0.89-0.94)
Positive tests	test	30-39	0.89 (0.86-0.91)
Positive tests	test	50-59	1.13 (1.10-1.16)
Positive tests	test	60-69	1.10 (1.07-1.13)
Positive tests	test	70-79	1.14 (1.11-1.17)
Positive tests	test	80+	1.32 (1.28-1.36)
Hospitalisations	population	0-9	0.24 (0.21-0.28)
Hospitalisations	population	10-19	0.14 (0.12-0.17)
Hospitalisations	population	0-29	0.34 (0.31-0.39)

Outcome	Denominator	Age group	IRR
Hospitalisations	population	30-39	0.50 (0.45-0.55)
Hospitalisations	population	50-59	2.03 (1.89-2.20)
Hospitalisations	population	60-69	4.00 (3.72-4.30)
Hospitalisations	population	70-79	7.79 (7.26-8.36)
Hospitalisations	population	80+	19.10 (17.82-20.54)
Hospitalisations	test	0-9	0.89 (0.76-1.03)
Hospitalisations	test	10-19	0.12 (0.10-0.15)
Hospitalisations	test	0-29	0.23 (0.20-0.26)
Hospitalisations	test	30-39	0.41 (0.36-0.45)
Hospitalisations	test	50-59	2.28 (2.11-2.48)
Hospitalisations	test	60-69	5.74 (5.33-6.20)
Hospitalisations	test	70-79	13.57 (12.62-14.66)
Hospitalisations	test	80+	20.49 (18.96-22.12)
Hospitalisations	positive test	0-9	1.64 (1.43-1.89)
Hospitalisations	positive test	10-19	0.17 (0.14-0.21)
Hospitalisations	positive test	0-29	0.28 (0.25-0.31)
Hospitalisations	positive test	30-39	0.48 (0.43-0.53)
Hospitalisations	positive test	50-59	1.97 (1.83-2.12)
Hospitalisations	positive test	60-69	4.75 (4.42-5.09)
Hospitalisations	positive test	70-79	10.39 (9.70-11.13)
Hospitalisations	positive test	80+	12.91 (12.07-13.82)
ICU admissions	population	50-59	7.36 (6.08-8.86)
ICU admissions	population	60-69	21.48 (18.17-25.59)
ICU admissions	population	70-79	40.81 (34.48-48.09)
ICU admissions	population	80+	38.17 (32.07-45.19)
ICU admissions	test	50-59	8.71 (6.98-10.90)
ICU admissions	test	60-69	32.03 (26.29-39.07)
ICU admissions	test	70-79	73.38 (60.56-89.39)
ICU admissions	test	80+	40.81 (33.11-50.58)
ICU admissions	positive test	50-59	6.14 (5.07-7.46)
ICU admissions	positive test	60-69	21.11 (17.82-25.16)
ICU admissions	positive test	70-79	44.28 (37.76-52.51)
ICU admissions	positive test	80+	20.92 (17.46-25.20)
Deaths	population	50-59	13.47 (9.92-18.74)
Deaths	population	60-69	68.62 (52.50-92.77)

Outcome	Denominator	Age group	IRR
Deaths	population	70-79	298.77 (229.59-401.71)
Deaths	population	80+	2059.46 (1581.27-2756.31)
Deaths	test	50-59	15.22 (10.66-21.88)
Deaths	test	60-69	93.17 (67.99-130.79)
Deaths	test	70-79	500.55 (368.55-690.49)
Deaths	test	80+	2096.71 (1544.63-2909.45)
Deaths	positive test	50-59	11.74 (8.59-16.05)
Deaths	positive test	60-69	71.77 (54.76-96.04)
Deaths	positive test	70-79	353.83 (272.29-470.17)
Deaths	positive test	80+	1174.64 (901.92-1554.92)

Table S7: Incidence rate ratios (95% credible interval) for the association between sex and five outcomes related to SARS-CoV-2 surveillance and care: total tests, positive tests, hospitalisations, intensive care unit (ICU) admissions and deaths. The reference group is males. The models are also adjusted for age, epidemic wave, canton and SEP decile.

Outcome	Denominator	Males	Females
Total tests	population	-	1.04 (1.02-1.06)
Positive tests	population	-	1.06 (1.04-1.08)
Positive tests	test	-	0.98 (0.97-0.99)
Hospitalisations	population	-	0.63 (0.61-0.66)
Hospitalisations	test	-	0.61 (0.59-0.64)
Hospitalisations	positive test	-	0.63 (0.61-0.65)
ICU admissions	population	-	0.33 (0.30-0.36)
ICU admissions	test	-	0.33 (0.29-0.36)
ICU admissions	positive test	-	0.34 (0.31-0.38)
Deaths	population	-	0.55 (0.51-0.59)
Deaths	test	-	0.54 (0.50-0.58)
Deaths	positive test	-	0.56 (0.53-0.59)

Table S8: Incidence rate ratios (95% credible interval) for the association between epidemic wave and five outcomes related to SARS-CoV-2 surveillance and care: total tests, positive tests, hospitalisations, intensive care unit (ICU) admissions and deaths. The reference group is before 8 June 2020. The models are also adjusted for age, sex, canton and SEP decile.

Outcome	Denominator	Before 8 June 2020	After 8 June 2020
Total tests	population	-	111.69 (109.35-114.02)
Positive tests	population	-	23.29 (22.69-23.91)
Positive tests	test	-	19.33 (17.12-21.87)
Hospitalisations	population	-	5.61 (5.38-5.86)
Hospitalisations	test	-	8.69 (6.20-12.46)
Hospitalisations	positive test	-	0.37 (0.35-0.39)
ICU admissions	population	-	3.58 (3.23-3.98)
ICU admissions	test	-	5.16 (2.40-14.12)
ICU admissions	positive test	-	0.25 (0.22-0.28)
Deaths	population	-	5.38 (4.93-5.84)
Deaths	test	-	19.05 (9.45-44.88)
Deaths	positive test	-	0.43 (0.40-0.46)

5.2 Interactions

Table S9: Estimates of interaction between SEP and five outcomes, separately for each covariate (corresponding to figure S10). For each combination of outcome, denominator and covariate are shown the interaction term (a value different than 1 indicates an interaction). Estimates correspond to median posterior and 95% credible interval The reference group for age is 40-49 (squares) for total tests, positive tests and hospitalisations. Because of the small number of events in younger groups, we pooled together age groups 0 to 49 for ICU admissions and deaths, and used this larger group as reference. The reference group is males and the first epidemic wave.

Outcome	Denominator	Covariate	Interaction term
Total tests	population	0-9	0.99 (0.98-1.01)
Total tests	population	10-19	0.99 (0.98-1.01)
Total tests	population	0-29	0.99 (0.98-1.00)
Total tests	population	30-39	1.00 (0.99-1.02)
Total tests	population	50-59	0.99 (0.98-1.00)
Total tests	population	60-69	0.98 (0.97-1.00)
Total tests	population	70-79	0.96 (0.95-0.98)
Total tests	population	80+	0.95 (0.94-0.97)
Total tests	population	Female	0.99 (0.99-1.00)

Outcome	Denominator	Covariate	Interaction term
Total tests	population	2nd wave	1.01 (1.00-1.01)
Positive tests	population	0-9	1.03 (1.01-1.04)
Positive tests	population	10-19	1.00 (0.98-1.02)
Positive tests	population	0-29	1.02 (1.00-1.04)
Positive tests	population	30-39	1.02 (1.00-1.03)
Positive tests	population	50-59	1.00 (0.98-1.01)
Positive tests	population	60-69	0.99 (0.97-1.00)
Positive tests	population	70-79	0.96 (0.95-0.98)
Positive tests	population	80+	0.96 (0.95-0.98)
Positive tests	population	Female	0.99 (0.98-1.00)
Positive tests	population	2nd wave	0.97 (0.97-0.98)
Positive tests	test	0-9	1.02 (1.01-1.04)
Positive tests	test	10-19	1.01 (1.00-1.02)
Positive tests	test	0-29	1.02 (1.01-1.03)
Positive tests	test	30-39	1.00 (1.00-1.01)
Positive tests	test	50-59	1.01 (1.00-1.02)
Positive tests	test	60-69	1.00 (0.99-1.01)
Positive tests	test	70-79	1.01 (1.00-1.02)
Positive tests	test	80+	1.03 (1.02-1.04)
Positive tests	test	Female	1.00 (0.99-1.00)
Positive tests	test	2nd wave	1.01 (0.97-1.05)
Hospitalisations	population	0-9	1.03 (0.98-1.09)
Hospitalisations	population	10-19	1.01 (0.96-1.08)
Hospitalisations	population	0-29	1.04 (0.99-1.08)
Hospitalisations	population	30-39	1.00 (0.96-1.03)
Hospitalisations	population	50-59	0.98 (0.95-1.01)
Hospitalisations	population	60-69	0.95 (0.93-0.98)
Hospitalisations	population	70-79	0.96 (0.94-0.99)
Hospitalisations	population	80+	1.00 (0.97-1.02)
Hospitalisations	population	Female	1.00 (0.98-1.01)
Hospitalisations	population	2nd wave	0.99 (0.97-1.00)
Hospitalisations	test	0-9	1.03 (0.98-1.09)
Hospitalisations	test	10-19	1.02 (0.95-1.09)
Hospitalisations	test	0-29	1.04 (0.99-1.09)
Hospitalisations	test	30-39	1.00 (0.96-1.04)

Outcome	Denominator	Covariate	Interaction term
Hospitalisations	test	50-59	1.00 (0.97-1.04)
Hospitalisations	test	60-69	0.97 (0.95-1.00)
Hospitalisations	test	70-79	1.00 (0.98-1.03)
Hospitalisations	test	80+	1.06 (1.03-1.09)
Hospitalisations	test	Female	1.00 (0.98-1.01)
Hospitalisations	test	2nd wave	0.98 (0.87-1.10)
Hospitalisations	positive test	0-9	1.00 (0.95-1.05)
Hospitalisations	positive test	10-19	1.01 (0.95-1.08)
Hospitalisations	positive test	0-29	1.02 (0.98-1.06)
Hospitalisations	positive test	30-39	0.99 (0.95-1.02)
Hospitalisations	positive test	50-59	0.98 (0.96-1.01)
Hospitalisations	positive test	60-69	0.96 (0.94-0.99)
Hospitalisations	positive test	70-79	0.99 (0.97-1.02)
Hospitalisations	positive test	80+	1.03 (1.01-1.06)
Hospitalisations	positive test	Female	1.00 (0.98-1.01)
Hospitalisations	positive test	2nd wave	0.99 (0.98-1.01)
ICU admissions	population	50-59	1.00 (0.93-1.06)
ICU admissions	population	60-69	1.03 (0.97-1.09)
ICU admissions	population	70-79	1.01 (0.95-1.07)
ICU admissions	population	80+	1.02 (0.96-1.09)
ICU admissions	population	Female	0.99 (0.96-1.03)
ICU admissions	population	2nd wave	0.99 (0.95-1.02)
ICU admissions	test	50-59	0.96 (0.89-1.04)
ICU admissions	test	60-69	1.01 (0.94-1.08)
ICU admissions	test	70-79	1.02 (0.95-1.10)
ICU admissions	test	80+	1.05 (0.98-1.14)
ICU admissions	test	Female	1.00 (0.96-1.04)
ICU admissions	test	2nd wave	0.96 (0.72-1.30)
ICU admissions	positive test	50-59	1.00 (0.94-1.07)
ICU admissions	positive test	60-69	1.04 (0.98-1.11)
ICU admissions	positive test	70-79	1.05 (0.99-1.11)
ICU admissions	positive test	80+	1.06 (1.00-1.13)
ICU admissions	positive test	Female	0.99 (0.96-1.03)
ICU admissions	positive test	2nd wave	0.99 (0.96-1.03)
Deaths	population	50-59	1.08 (0.96-1.23)

Outcome	Denominator	Covariate	Interaction term
Deaths	population	60-69	1.09 (0.98-1.22)
Deaths	population	70-79	1.08 (0.97-1.21)
Deaths	population	80+	1.14 (1.03-1.28)
Deaths	population	Female	1.03 (1.00-1.06)
Deaths	population	2nd wave	1.02 (0.99-1.05)
Deaths	test	50-59	1.09 (0.95-1.24)
Deaths	test	60-69	1.08 (0.96-1.24)
Deaths	test	70-79	1.09 (0.97-1.23)
Deaths	test	80+	1.18 (1.05-1.34)
Deaths	test	Female	1.03 (1.00-1.05)
Deaths	test	2nd wave	1.31 (0.97-1.94)
Deaths	positive test	50-59	1.10 (0.97-1.25)
Deaths	positive test	60-69	1.11 (1.00-1.25)
Deaths	positive test	70-79	1.12 (1.01-1.25)
Deaths	positive test	80+	1.18 (1.07-1.33)
Deaths	positive test	Female	1.02 (1.00-1.03)
Deaths	positive test	2nd wave	1.02 (0.99-1.04)

Table S10: Estimates of interaction between SEP and five outcomes, separately for each canton (corresponding to figure S8). For each combination of outcome, denominator and canton are shown the interaction term (a value different than 1 indicates an interaction) and the incidence rate ratio (computed by multiplying the interaction term by the average effect). Estimates correspond to median posterior and 95% credible interval.

Outcome	Denominator	Canton	Interaction term	IRR
Total tests	population	AG	0.99 (0.97-1.01)	1.01 (0.99-1.02)
Positive tests	population	AG	0.97 (0.95-0.99)	0.97 (0.95-0.98)
Positive tests	test	AG	0.99 (0.98-1.01)	0.96 (0.95-0.97)
Hospitalisations	population	AG	0.98 (0.95-1.01)	0.92 (0.89-0.94)
Hospitalisations	test	AG	1.00 (0.97-1.03)	0.92 (0.90-0.95)
Hospitalisations	positive test	AG	1.00 (0.98-1.01)	0.96 (0.94-0.97)
ICU admissions	population	AG	1.00 (0.94-1.06)	0.90 (0.85-0.95)
ICU admissions	test	AG	1.01 (0.96-1.07)	0.91 (0.87-0.96)
ICU admissions	positive test	AG	1.01 (0.96-1.06)	0.94 (0.90-0.99)
Deaths	population	AG	0.96 (0.90-1.03)	0.93 (0.88-0.98)
Deaths	test	AG	1.00 (0.97-1.04)	0.96 (0.93-1.00)

Outcome	Denominator	Canton	Interaction term	IRR
Deaths	positive test	AG	1.00 (0.97-1.03)	0.98 (0.96-1.01)
Total tests	population	AI	1.05 (1.01-1.08)	1.07 (1.04-1.10)
Positive tests	population	AI	1.07 (1.03-1.12)	1.07 (1.03-1.11)
Positive tests	test	AI	1.03 (1.00-1.06)	0.99 (0.97-1.02)
Hospitalisations	population	AI	1.02 (0.96-1.10)	0.96 (0.90-1.03)
Hospitalisations	test	AI	1.00 (0.95-1.06)	0.92 (0.88-0.98)
Hospitalisations	positive test	AI	1.00 (0.98-1.03)	0.96 (0.93-0.99)
ICU admissions	population	AI	0.99 (0.87-1.11)	0.89 (0.78-1.01)
ICU admissions	test	AI	1.00 (0.91-1.09)	0.90 (0.82-0.99)
ICU admissions	positive test	AI	1.00 (0.92-1.08)	0.94 (0.86-1.02)
Deaths	population	AI	1.12 (0.96-1.33)	1.08 (0.92-1.28)
Deaths	test	AI	1.01 (0.96-1.09)	0.96 (0.92-1.04)
Deaths	positive test	AI	1.01 (0.98-1.07)	1.00 (0.96-1.05)
Total tests	population	AR	0.99 (0.97-1.02)	1.01 (0.99-1.03)
Positive tests	population	AR	1.01 (0.98-1.03)	1.00 (0.98-1.02)
Positive tests	test	AR	1.02 (1.00-1.03)	0.98 (0.97-1.00)
Hospitalisations	population	AR	1.02 (0.97-1.08)	0.96 (0.91-1.01)
Hospitalisations	test	AR	1.02 (0.97-1.07)	0.94 (0.90-0.99)
Hospitalisations	positive test	AR	1.00 (0.98-1.03)	0.96 (0.94-0.99)
ICU admissions	population	AR	1.02 (0.93-1.12)	0.92 (0.84-1.01)
ICU admissions	test	AR	1.01 (0.94-1.12)	0.91 (0.85-1.01)
ICU admissions	positive test	AR	1.00 (0.94-1.09)	0.94 (0.87-1.02)
Deaths	population	AR	0.98 (0.87-1.09)	0.94 (0.85-1.05)
Deaths	test	AR	1.00 (0.95-1.06)	0.96 (0.91-1.02)
Deaths	positive test	AR	1.00 (0.95-1.04)	0.98 (0.94-1.02)
Total tests	population	BE	1.05 (1.03-1.07)	1.07 (1.05-1.08)
Positive tests	population	BE	1.00 (0.98-1.02)	0.99 (0.98-1.01)
Positive tests	test	BE	0.97 (0.96-0.98)	0.94 (0.93-0.95)
Hospitalisations	population	BE	0.98 (0.95-1.01)	0.92 (0.90-0.94)
Hospitalisations	test	BE	0.97 (0.95-1.00)	0.90 (0.88-0.92)
Hospitalisations	positive test	BE	0.99 (0.97-1.01)	0.95 (0.93-0.96)
ICU admissions	population	BE	0.99 (0.94-1.04)	0.89 (0.85-0.93)
ICU admissions	test	BE	0.99 (0.94-1.03)	0.89 (0.85-0.93)
ICU admissions	positive test	BE	0.99 (0.94-1.03)	0.92 (0.88-0.96)
Deaths	population	BE	0.98 (0.91-1.04)	0.94 (0.90-0.99)

Outcome	Denominator	Canton	Interaction term	IRR
Deaths	test	BE	1.00 (0.97-1.03)	0.96 (0.93-0.99)
Deaths	positive test	BE	1.00 (0.98-1.03)	0.99 (0.96-1.01)
Total tests	population	BL	0.96 (0.94-0.98)	0.98 (0.97-0.99)
Positive tests	population	BL	0.97 (0.94-0.99)	0.96 (0.94-0.98)
Positive tests	test	BL	1.00 (0.99-1.01)	0.97 (0.96-0.98)
Hospitalisations	population	BL	0.97 (0.93-1.00)	0.91 (0.88-0.93)
Hospitalisations	test	BL	1.02 (0.99-1.05)	0.94 (0.92-0.97)
Hospitalisations	positive test	BL	1.00 (0.99-1.03)	0.96 (0.95-0.99)
ICU admissions	population	BL	0.95 (0.88-1.02)	0.86 (0.80-0.91)
ICU admissions	test	BL	0.99 (0.91-1.04)	0.89 (0.82-0.95)
ICU admissions	positive test	BL	0.99 (0.92-1.05)	0.93 (0.86-0.98)
Deaths	population	BL	0.87 (0.80-0.94)	0.84 (0.79-0.90)
Deaths	test	BL	0.98 (0.93-1.02)	0.94 (0.89-0.98)
Deaths	positive test	BL	1.00 (0.95-1.02)	0.98 (0.94-1.01)
Total tests	population	BS	0.99 (0.97-1.01)	1.00 (0.99-1.02)
Positive tests	population	BS	1.01 (0.99-1.03)	1.00 (0.99-1.02)
Positive tests	test	BS	0.98 (0.96-0.99)	0.95 (0.93-0.96)
Hospitalisations	population	BS	0.98 (0.95-1.01)	0.92 (0.89-0.95)
Hospitalisations	test	BS	0.97 (0.94-1.00)	0.90 (0.87-0.93)
Hospitalisations	positive test	BS	1.00 (0.97-1.01)	0.95 (0.93-0.97)
ICU admissions	population	BS	0.95 (0.87-1.02)	0.85 (0.78-0.91)
ICU admissions	test	BS	0.97 (0.87-1.03)	0.87 (0.79-0.93)
ICU admissions	positive test	BS	0.97 (0.89-1.02)	0.90 (0.83-0.95)
Deaths	population	BS	0.94 (0.86-1.01)	0.90 (0.84-0.97)
Deaths	test	BS	0.98 (0.92-1.02)	0.94 (0.88-0.97)
Deaths	positive test	BS	0.98 (0.94-1.01)	0.97 (0.93-0.99)
Total tests	population	FR	0.98 (0.96-1.01)	1.00 (0.99-1.02)
Positive tests	population	FR	0.98 (0.96-1.00)	0.97 (0.96-0.99)
Positive tests	test	FR	1.01 (0.99-1.02)	0.98 (0.97-0.99)
Hospitalisations	population	FR	1.02 (0.98-1.05)	0.95 (0.93-0.98)
Hospitalisations	test	FR	1.03 (1.00-1.07)	0.95 (0.92-0.98)
Hospitalisations	positive test	FR	1.01 (0.99-1.04)	0.97 (0.95-1.00)
ICU admissions	population	FR	1.09 (1.02-1.17)	0.98 (0.92-1.05)
ICU admissions	test	FR	1.04 (0.98-1.14)	0.94 (0.88-1.02)
ICU admissions	positive test	FR	1.05 (0.99-1.12)	0.98 (0.92-1.05)

Outcome	Denominator	Canton	Interaction term	IRR
Deaths	population	FR	0.96 (0.89-1.04)	0.93 (0.87-0.99)
Deaths	test	FR	1.00 (0.95-1.04)	0.95 (0.92-0.99)
Deaths	positive test	FR	1.00 (0.97-1.03)	0.98 (0.95-1.01)
Total tests	population	GE	1.09 (1.07-1.11)	1.11 (1.09-1.12)
Positive tests	population	GE	1.08 (1.05-1.10)	1.07 (1.06-1.09)
Positive tests	test	GE	0.98 (0.97-1.00)	0.95 (0.94-0.96)
Hospitalisations	population	GE	1.06 (1.03-1.09)	0.99 (0.97-1.02)
Hospitalisations	test	GE	1.01 (0.98-1.03)	0.93 (0.90-0.95)
Hospitalisations	positive test	GE	1.00 (0.99-1.02)	0.96 (0.94-0.98)
ICU admissions	population	GE	1.10 (1.05-1.17)	0.99 (0.95-1.04)
ICU admissions	test	GE	1.01 (0.97-1.07)	0.91 (0.87-0.96)
ICU admissions	positive test	GE	1.02 (0.98-1.07)	0.95 (0.92-0.99)
Deaths	population	GE	1.08 (1.01-1.15)	1.04 (0.99-1.09)
Deaths	test	GE	1.00 (0.97-1.04)	0.96 (0.93-1.00)
Deaths	positive test	GE	1.01 (0.98-1.04)	0.99 (0.97-1.02)
Total tests	population	GL	1.00 (0.97-1.03)	1.02 (0.99-1.04)
Positive tests	population	GL	1.03 (1.00-1.07)	1.03 (1.00-1.06)
Positive tests	test	GL	1.02 (1.00-1.04)	0.99 (0.97-1.01)
Hospitalisations	population	GL	1.02 (0.96-1.08)	0.95 (0.90-1.01)
Hospitalisations	test	GL	1.00 (0.95-1.04)	0.92 (0.88-0.97)
Hospitalisations	positive test	GL	0.99 (0.96-1.02)	0.95 (0.92-0.97)
ICU admissions	population	GL	1.06 (0.96-1.19)	0.95 (0.86-1.07)
ICU admissions	test	GL	1.02 (0.95-1.14)	0.92 (0.85-1.03)
ICU admissions	positive test	GL	1.00 (0.92-1.09)	0.93 (0.85-1.02)
Deaths	population	GL	1.09 (0.96-1.23)	1.05 (0.92-1.18)
Deaths	test	GL	1.00 (0.96-1.07)	0.96 (0.91-1.03)
Deaths	positive test	GL	0.98 (0.93-1.02)	0.96 (0.92-1.01)
Total tests	population	GR	1.01 (0.99-1.03)	1.03 (1.02-1.05)
Positive tests	population	GR	0.98 (0.95-1.00)	0.97 (0.95-0.99)
Positive tests	test	GR	1.00 (0.98-1.01)	0.97 (0.96-0.98)
Hospitalisations	population	GR	1.01 (0.98-1.05)	0.95 (0.92-0.99)
Hospitalisations	test	GR	1.01 (0.97-1.04)	0.93 (0.90-0.96)
Hospitalisations	positive test	GR	1.00 (0.99-1.03)	0.96 (0.94-0.99)
ICU admissions	population	GR	0.97 (0.89-1.05)	0.87 (0.80-0.95)
ICU admissions	test	GR	0.99 (0.91-1.07)	0.90 (0.82-0.98)

Outcome	Denominator	Canton	Interaction term	IRR
ICU admissions	positive test	GR	0.99 (0.91-1.05)	0.92 (0.85-0.98)
Deaths	population	GR	1.00 (0.91-1.08)	0.96 (0.89-1.04)
Deaths	test	GR	1.00 (0.96-1.07)	0.96 (0.92-1.02)
Deaths	positive test	GR	1.01 (0.98-1.05)	0.99 (0.97-1.04)
Total tests	population	JU	0.97 (0.94-0.99)	0.99 (0.96-1.01)
Positive tests	population	JU	1.03 (1.00-1.05)	1.02 (0.99-1.05)
Positive tests	test	JU	1.01 (1.00-1.03)	0.98 (0.96-1.00)
Hospitalisations	population	JU	1.03 (0.97-1.09)	0.96 (0.91-1.02)
Hospitalisations	test	JU	1.01 (0.96-1.06)	0.93 (0.89-0.98)
Hospitalisations	positive test	JU	1.01 (0.98-1.03)	0.96 (0.94-0.99)
ICU admissions	population	JU	1.01 (0.91-1.14)	0.91 (0.82-1.03)
ICU admissions	test	JU	1.00 (0.91-1.09)	0.90 (0.82-0.99)
ICU admissions	positive test	JU	1.00 (0.93-1.09)	0.94 (0.87-1.02)
Deaths	population	JU	1.01 (0.88-1.15)	0.97 (0.85-1.10)
Deaths	test	JU	0.99 (0.92-1.05)	0.95 (0.88-1.01)
Deaths	positive test	JU	1.03 (0.98-1.09)	1.02 (0.97-1.07)
Total tests	population	LU	1.01 (0.99-1.04)	1.03 (1.02-1.05)
Positive tests	population	LU	0.99 (0.97-1.01)	0.99 (0.97-1.00)
Positive tests	test	LU	0.97 (0.96-0.98)	0.94 (0.93-0.95)
Hospitalisations	population	LU	0.97 (0.94-1.00)	0.91 (0.89-0.94)
Hospitalisations	test	LU	0.97 (0.94-1.00)	0.90 (0.87-0.92)
Hospitalisations	positive test	LU	1.00 (0.97-1.01)	0.95 (0.93-0.97)
ICU admissions	population	LU	0.99 (0.93-1.06)	0.89 (0.84-0.95)
ICU admissions	test	LU	1.00 (0.94-1.06)	0.90 (0.85-0.95)
ICU admissions	positive test	LU	1.00 (0.95-1.06)	0.94 (0.88-0.99)
Deaths	population	LU	0.97 (0.90-1.04)	0.93 (0.88-0.99)
Deaths	test	LU	0.99 (0.93-1.02)	0.94 (0.90-0.97)
Deaths	positive test	LU	1.00 (0.97-1.03)	0.99 (0.96-1.02)
Total tests	population	NE	0.96 (0.94-0.98)	0.98 (0.96-1.00)
Positive tests	population	NE	1.00 (0.97-1.02)	0.99 (0.97-1.01)
Positive tests	test	NE	1.02 (1.01-1.04)	0.99 (0.98-1.00)
Hospitalisations	population	NE	0.99 (0.95-1.03)	0.93 (0.89-0.97)
Hospitalisations	test	NE	1.01 (0.97-1.06)	0.94 (0.90-0.98)
Hospitalisations	positive test	NE	1.00 (0.97-1.03)	0.96 (0.93-0.98)
ICU admissions	population	NE	1.03 (0.95-1.11)	0.92 (0.85-1.00)

Outcome	Denominator	Canton	Interaction term	IRR
ICU admissions	test	NE	1.00 (0.94-1.09)	0.91 (0.85-0.98)
ICU admissions	positive test	NE	1.01 (0.95-1.08)	0.94 (0.89-1.01)
Deaths	population	NE	1.00 (0.92-1.08)	0.96 (0.89-1.03)
Deaths	test	NE	1.01 (0.97-1.06)	0.96 (0.92-1.01)
Deaths	positive test	NE	1.00 (0.97-1.04)	0.98 (0.96-1.02)
Total tests	population	NW	1.00 (0.98-1.03)	1.02 (1.00-1.04)
Positive tests	population	NW	0.99 (0.96-1.01)	0.98 (0.96-1.01)
Positive tests	test	NW	1.00 (0.99-1.02)	0.97 (0.96-0.99)
Hospitalisations	population	NW	1.01 (0.96-1.06)	0.95 (0.90-1.00)
Hospitalisations	test	NW	1.00 (0.96-1.05)	0.92 (0.88-0.97)
Hospitalisations	positive test	NW	1.00 (0.98-1.03)	0.96 (0.94-0.99)
ICU admissions	population	NW	0.97 (0.87-1.08)	0.87 (0.78-0.98)
ICU admissions	test	NW	1.00 (0.93-1.10)	0.91 (0.84-1.00)
ICU admissions	positive test	NW	1.01 (0.93-1.10)	0.94 (0.87-1.04)
Deaths	population	NW	0.97 (0.84-1.11)	0.93 (0.81-1.07)
Deaths	test	NW	1.00 (0.95-1.06)	0.96 (0.91-1.02)
Deaths	positive test	NW	1.01 (0.97-1.07)	1.00 (0.95-1.06)
Total tests	population	OW	1.07 (1.04-1.09)	1.09 (1.06-1.11)
Positive tests	population	OW	1.04 (1.01-1.07)	1.03 (1.00-1.06)
Positive tests	test	OW	0.99 (0.97-1.01)	0.96 (0.94-0.98)
Hospitalisations	population	OW	1.02 (0.97-1.09)	0.96 (0.91-1.02)
Hospitalisations	test	OW	0.99 (0.94-1.03)	0.91 (0.86-0.95)
Hospitalisations	positive test	OW	1.00 (0.97-1.02)	0.95 (0.93-0.98)
ICU admissions	population	OW	0.98 (0.87-1.12)	0.88 (0.78-1.02)
ICU admissions	test	OW	1.00 (0.93-1.10)	0.91 (0.83-1.00)
ICU admissions	positive test	OW	1.01 (0.94-1.11)	0.94 (0.87-1.04)
Deaths	population	OW	1.36 (1.18-1.59)	1.32 (1.13-1.54)
Deaths	test	OW	1.02 (0.99-1.12)	0.98 (0.94-1.08)
Deaths	positive test	OW	1.01 (0.98-1.08)	1.00 (0.96-1.06)
Total tests	population	SG	0.97 (0.95-0.99)	0.99 (0.97-1.00)
Positive tests	population	SG	1.01 (0.99-1.03)	1.01 (0.99-1.02)
Positive tests	test	SG	1.03 (1.01-1.04)	0.99 (0.98-1.00)
Hospitalisations	population	SG	1.01 (0.98-1.04)	0.95 (0.93-0.97)
Hospitalisations	test	SG	1.02 (0.99-1.05)	0.94 (0.92-0.96)
Hospitalisations	positive test	SG	1.00 (0.99-1.02)	0.96 (0.95-0.98)

Outcome	Denominator	Canton	Interaction term	IRR
ICU admissions	population	SG	1.02 (0.96-1.09)	0.92 (0.87-0.97)
ICU admissions	test	SG	1.01 (0.95-1.07)	0.91 (0.86-0.96)
ICU admissions	positive test	SG	1.01 (0.96-1.07)	0.94 (0.90-1.00)
Deaths	population	SG	0.99 (0.93-1.06)	0.96 (0.91-1.01)
Deaths	test	SG	1.01 (0.97-1.06)	0.97 (0.93-1.01)
Deaths	positive test	SG	0.99 (0.97-1.02)	0.98 (0.95-1.01)
Total tests	population	SH	0.99 (0.97-1.01)	1.01 (0.99-1.03)
Positive tests	population	SH	0.97 (0.95-1.00)	0.97 (0.95-0.99)
Positive tests	test	SH	0.98 (0.97-1.00)	0.95 (0.94-0.96)
Hospitalisations	population	SH	1.00 (0.96-1.05)	0.94 (0.90-0.99)
Hospitalisations	test	SH	1.01 (0.97-1.05)	0.93 (0.90-0.97)
Hospitalisations	positive test	SH	1.00 (0.98-1.03)	0.96 (0.94-0.99)
ICU admissions	population	SH	0.98 (0.88-1.07)	0.88 (0.80-0.97)
ICU admissions	test	SH	1.00 (0.92-1.08)	0.90 (0.83-0.98)
ICU admissions	positive test	SH	1.00 (0.93-1.08)	0.94 (0.86-1.02)
Deaths	population	SH	0.95 (0.86-1.05)	0.92 (0.83-1.01)
Deaths	test	SH	1.00 (0.94-1.04)	0.95 (0.90-1.00)
Deaths	positive test	SH	0.99 (0.95-1.03)	0.97 (0.93-1.01)
Total tests	population	SO	1.00 (0.98-1.02)	1.02 (1.00-1.04)
Positive tests	population	SO	0.98 (0.96-1.00)	0.98 (0.96-0.99)
Positive tests	test	SO	0.97 (0.96-0.99)	0.94 (0.93-0.95)
Hospitalisations	population	SO	0.97 (0.93-1.00)	0.91 (0.88-0.93)
Hospitalisations	test	SO	0.97 (0.94-1.00)	0.90 (0.87-0.92)
Hospitalisations	positive test	SO	0.99 (0.97-1.01)	0.95 (0.93-0.97)
ICU admissions	population	SO	0.95 (0.87-1.02)	0.86 (0.79-0.91)
ICU admissions	test	SO	0.97 (0.89-1.03)	0.88 (0.81-0.93)
ICU admissions	positive test	SO	0.97 (0.89-1.02)	0.90 (0.83-0.96)
Deaths	population	SO	0.96 (0.89-1.04)	0.93 (0.87-0.99)
Deaths	test	SO	1.00 (0.95-1.04)	0.95 (0.92-0.99)
Deaths	positive test	SO	1.00 (0.97-1.03)	0.98 (0.95-1.02)
Total tests	population	SZ	1.00 (0.98-1.03)	1.02 (1.01-1.04)
Positive tests	population	SZ	0.98 (0.96-1.01)	0.98 (0.96-1.00)
Positive tests	test	SZ	0.98 (0.97-1.00)	0.95 (0.94-0.96)
Hospitalisations	population	SZ	0.99 (0.95-1.02)	0.92 (0.90-0.96)
Hospitalisations	test	SZ	0.99 (0.95-1.02)	0.91 (0.88-0.94)

Outcome	Denominator	Canton	Interaction term	IRR
Hospitalisations	positive test	SZ	1.00 (0.98-1.02)	0.96 (0.94-0.98)
ICU admissions	population	SZ	1.01 (0.93-1.09)	0.91 (0.85-0.98)
ICU admissions	test	SZ	0.99 (0.91-1.05)	0.89 (0.83-0.95)
ICU admissions	positive test	SZ	1.00 (0.93-1.06)	0.93 (0.87-0.99)
Deaths	population	SZ	0.94 (0.86-1.02)	0.91 (0.84-0.97)
Deaths	test	SZ	0.99 (0.94-1.03)	0.95 (0.90-0.99)
Deaths	positive test	SZ	0.99 (0.95-1.02)	0.97 (0.93-1.00)
Total tests	population	TG	0.97 (0.95-0.99)	0.99 (0.97-1.00)
Positive tests	population	TG	0.97 (0.95-0.99)	0.97 (0.95-0.98)
Positive tests	test	TG	1.00 (0.99-1.02)	0.97 (0.96-0.98)
Hospitalisations	population	TG	0.95 (0.92-0.99)	0.90 (0.86-0.92)
Hospitalisations	test	TG	0.99 (0.96-1.02)	0.92 (0.89-0.94)
Hospitalisations	positive test	TG	0.99 (0.97-1.01)	0.95 (0.92-0.97)
ICU admissions	population	TG	0.99 (0.92-1.06)	0.89 (0.83-0.95)
ICU admissions	test	TG	1.01 (0.95-1.08)	0.91 (0.86-0.98)
ICU admissions	positive test	TG	0.99 (0.93-1.05)	0.93 (0.87-0.98)
Deaths	population	TG	0.90 (0.83-0.97)	0.87 (0.81-0.93)
Deaths	test	TG	0.99 (0.94-1.03)	0.95 (0.90-0.98)
Deaths	positive test	TG	0.96 (0.92-1.00)	0.95 (0.91-0.98)
Total tests	population	TI	0.99 (0.97-1.01)	1.01 (1.00-1.03)
Positive tests	population	TI	0.95 (0.92-0.97)	0.94 (0.92-0.96)
Positive tests	test	TI	1.01 (0.99-1.02)	0.98 (0.96-0.99)
Hospitalisations	population	TI	0.98 (0.95-1.01)	0.92 (0.90-0.94)
Hospitalisations	test	TI	1.00 (0.98-1.03)	0.93 (0.90-0.95)
Hospitalisations	positive test	TI	1.00 (0.98-1.02)	0.96 (0.94-0.98)
ICU admissions	population	TI	1.01 (0.94-1.08)	0.91 (0.85-0.97)
ICU admissions	test	TI	1.00 (0.94-1.06)	0.90 (0.85-0.95)
ICU admissions	positive test	TI	1.00 (0.95-1.05)	0.94 (0.89-0.98)
Deaths	population	TI	0.91 (0.84-0.98)	0.87 (0.82-0.93)
Deaths	test	TI	0.99 (0.94-1.03)	0.95 (0.90-0.98)
Deaths	positive test	TI	0.99 (0.95-1.01)	0.97 (0.94-0.99)
Total tests	population	UR	1.06 (1.03-1.09)	1.08 (1.05-1.10)
Positive tests	population	UR	1.06 (1.03-1.09)	1.05 (1.02-1.09)
Positive tests	test	UR	1.01 (0.99-1.03)	0.98 (0.96-1.00)
Hospitalisations	population	UR	1.04 (0.99-1.12)	0.98 (0.92-1.05)

Outcome	Denominator	Canton	Interaction term	IRR
Hospitalisations	test	UR	1.01 (0.96-1.06)	0.93 (0.88-0.98)
Hospitalisations	positive test	UR	1.00 (0.98-1.03)	0.96 (0.94-0.98)
ICU admissions	population	UR	0.97 (0.85-1.09)	0.87 (0.76-0.99)
ICU admissions	test	UR	1.00 (0.90-1.08)	0.90 (0.81-0.98)
ICU admissions	positive test	UR	1.00 (0.92-1.08)	0.93 (0.85-1.02)
Deaths	population	UR	1.19 (1.05-1.35)	1.15 (1.01-1.30)
Deaths	test	UR	1.01 (0.97-1.09)	0.97 (0.93-1.05)
Deaths	positive test	UR	1.00 (0.97-1.05)	0.99 (0.95-1.03)
Total tests	population	VD	0.97 (0.95-0.99)	0.99 (0.97-1.00)
Positive tests	population	VD	1.00 (0.98-1.02)	1.00 (0.98-1.01)
Positive tests	test	VD	1.01 (1.00-1.03)	0.98 (0.97-0.99)
Hospitalisations	population	VD	1.01 (0.98-1.03)	0.95 (0.93-0.96)
Hospitalisations	test	VD	1.01 (0.98-1.03)	0.93 (0.91-0.95)
Hospitalisations	positive test	VD	1.00 (0.99-1.02)	0.96 (0.95-0.97)
ICU admissions	population	VD	1.00 (0.95-1.06)	0.90 (0.86-0.94)
ICU admissions	test	VD	1.00 (0.95-1.05)	0.90 (0.86-0.95)
ICU admissions	positive test	VD	0.99 (0.94-1.03)	0.92 (0.88-0.96)
Deaths	population	VD	1.00 (0.94-1.07)	0.97 (0.93-1.01)
Deaths	test	VD	1.00 (0.96-1.03)	0.95 (0.92-0.99)
Deaths	positive test	VD	1.01 (0.99-1.03)	0.99 (0.97-1.02)
Total tests	population	VS	0.98 (0.96-1.00)	1.00 (0.98-1.02)
Positive tests	population	VS	1.00 (0.98-1.03)	1.00 (0.98-1.02)
Positive tests	test	VS	1.03 (1.01-1.04)	0.99 (0.98-1.01)
Hospitalisations	population	VS	1.04 (1.01-1.09)	0.98 (0.94-1.02)
Hospitalisations	test	VS	1.04 (1.00-1.08)	0.96 (0.92-1.00)
Hospitalisations	positive test	VS	1.01 (0.99-1.04)	0.97 (0.95-1.00)
ICU admissions	population	VS	1.06 (0.96-1.19)	0.95 (0.86-1.07)
ICU admissions	test	VS	1.03 (0.97-1.16)	0.93 (0.87-1.05)
ICU admissions	positive test	VS	1.05 (0.97-1.16)	0.98 (0.90-1.08)
Deaths	population	VS	1.03 (0.95-1.12)	1.00 (0.92-1.07)
Deaths	test	VS	1.01 (0.98-1.08)	0.97 (0.94-1.04)
Deaths	positive test	VS	1.01 (0.98-1.04)	0.99 (0.97-1.03)
Total tests	population	ZG	0.99 (0.97-1.01)	1.01 (1.00-1.03)
Positive tests	population	ZG	0.98 (0.95-1.00)	0.97 (0.95-0.99)
Positive tests	test	ZG	0.99 (0.98-1.01)	0.96 (0.95-0.97)

Outcome	Denominator	Canton	Interaction term	IRR
Hospitalisations	population	ZG	0.97 (0.93-1.02)	0.91 (0.87-0.95)
Hospitalisations	test	ZG	0.98 (0.94-1.02)	0.91 (0.87-0.94)
Hospitalisations	positive test	ZG	1.00 (0.97-1.02)	0.96 (0.93-0.98)
ICU admissions	population	ZG	0.93 (0.84-1.02)	0.84 (0.76-0.92)
ICU admissions	test	ZG	0.98 (0.88-1.06)	0.89 (0.79-0.96)
ICU admissions	positive test	ZG	0.98 (0.90-1.05)	0.92 (0.84-0.99)
Deaths	population	ZG	1.03 (0.93-1.14)	0.99 (0.90-1.09)
Deaths	test	ZG	1.00 (0.96-1.06)	0.96 (0.92-1.02)
Deaths	positive test	ZG	1.01 (0.98-1.06)	1.00 (0.96-1.05)
Total tests	population	ZH	0.97 (0.95-0.99)	0.99 (0.98-1.01)
Positive tests	population	ZH	0.97 (0.95-0.99)	0.97 (0.95-0.98)
Positive tests	test	ZH	0.99 (0.98-1.00)	0.96 (0.95-0.97)
Hospitalisations	population	ZH	0.96 (0.93-0.98)	0.90 (0.88-0.92)
Hospitalisations	test	ZH	0.99 (0.96-1.01)	0.91 (0.89-0.93)
Hospitalisations	positive test	ZH	0.99 (0.97-1.00)	0.95 (0.93-0.96)
ICU admissions	population	ZH	0.97 (0.93-1.02)	0.88 (0.84-0.91)
ICU admissions	test	ZH	0.99 (0.94-1.03)	0.89 (0.85-0.92)
ICU admissions	positive test	ZH	0.99 (0.95-1.03)	0.92 (0.89-0.96)
Deaths	population	ZH	0.93 (0.87-0.99)	0.89 (0.85-0.93)
Deaths	test	ZH	0.98 (0.93-1.01)	0.94 (0.90-0.96)
Deaths	positive test	ZH	0.98 (0.95-1.01)	0.97 (0.94-0.99)

References

[1] Goodrich B, Gabry J, Ali I. Brilleman, S. rstanarm: Bayesian Applied Regression Modeling Via Stan. R Package Version 2.21.1; 2020.

[2] Gabry J, Goodrich B. Estimating Generalized (Non-)Linear Models with Group-Specific Terms with rstanarm; 2020. http://mc-stan.org/rstanarm/articles/glmer.html.

[3] Vehtari A, Gelman A, Gabry J. Practical Bayesian model evaluation using leave-one-out cross-validation and WAIC. Statistics and computing. 2017;27(5):1413–1432.

[4] Google web services, geocoding API; 2020. https://developers.google.com/maps/documentation/geocoding/overview.
